# A general framework for modeling pathogen transmission in co‐roosting host communities

**DOI:** 10.1002/ecy.70326

**Published:** 2026-02-20

**Authors:** Molly C. Simonis, Daniel J. Becker

**Affiliations:** ^1^ School of Biological Sciences, University of Oklahoma Norman Oklahoma USA; ^2^ College of Forestry, Wildlife, and Environment and College of Veterinary Medicine, Department of Pathobiology Auburn University Auburn Alabama USA

**Keywords:** bats, cross‐species transmission, disease ecology, epidemiological models, multi‐host transmission, phylogenetic similarity

## Abstract

Cross‐species transmission of pathogens can be facilitated by frequent contact among wildlife. Cross‐species transmission is often driven by phylogenetic similarity between host species, but the role this plays when multiple host species co‐roost is unknown. We developed a generalizable framework for understanding how cross‐species transmission is driven by contact among co‐roosting species spanning evolutionary similarities and the net impact on roost‐level infection prevalence. We developed ordinary differential equation models describing population and infection dynamics between two and three co‐roosting species. We derived conditions for pathogen invasion and parameterized models using co‐roosting Neotropical bat systems, with interspecific transmission exponentially declining with phylogenetic distance. To assess the relative contribution of contact rates and phylogenetic similarity, we co‐varied intraspecific transmission rates and phylogenetic distances while considering sensitivity to epidemiological structure and pathogen traits. For both susceptible–infected–recovered–susceptible and susceptible–infected–latent–infected models, we show that relatedness between co‐roosting hosts facilitates pathogen invasion, particularly for poorly transmissible pathogens with short durations of infection and immunity or latency. These models converged on similar equilibria, and roost‐level prevalence was greatest when hosts were most closely related. However, we also identified regions of parameter space where roost‐level prevalence increased when hosts were distantly related. Our generalizable models are adaptable to other co‐roosting systems with low‐virulence pathogens that are directly transmitted and inform our understanding of pathogen spillover.

## INTRODUCTION

Zoonotic pathogens, which spillover from wildlife to humans, are one of the largest threats to human health (Daszak, 2000; Jones et al., 2008). In recent years, we have seen multiple deadly epidemics (and at least two pandemics) of emerging infectious diseases that were a result of zoonotic spillover (e.g., Nipah virus, Ebola virus, Hendra virus, Marburg virus, H1N1 influenza virus, MERS‐CoV, SARS‐CoV, and SARS‐CoV‐2; Piret & Boivin, 2021). Inter‐species contact and susceptibility to infection are required for cross‐species transmission, with the latter formed by coevolutionary immunological barriers between hosts and their pathogens (Combes, 2001). As such, cross‐species transmission is more common for closely related host species (Albery et al., 2021; Faria et al., 2013; Huang et al., 2014; Longdon et al., 2011; Pedersen & Davies, 2009; Parker et al., 2015; Streicker et al., 2012; Willoughby et al. 2017). However, the role phylogenetic similarity plays when multiple host species have regular and epidemiologically relevant contacts, such as what occurs within shared roosts, is less understood.

Co‐roosting (i.e., multiple species sharing a roost) is common across wildlife taxa and can facilitate cross‐species transmission due to close interactions between hosts (Kreuder Johnson et al., 2015; Reluga et al., 2007). There are many examples of co‐roosting in natural and anthropogenic systems (Table 1). Many meso‐ and top carnivores are known to share dens (Hendrickson et al., 2005; Kondo, 2018; Shirer & Fitch, 1970), and multiple bird species can share natural and anthropogenic cavities (Finch, 1990; Lammertink et al., 2019; Pearce & Foote, 2022; Peer et al., 2001). Even though co‐roosting and overall gregariousness are strong predictors of viral sharing among species at macroecological scales (Luis et al., 2015; Willoughby et al., 2017), few studies present co‐roosting as facilitating pathogen transmission (but see Hoyt et al., 2018, Soupé‐Gilbert et al., 2022). However, growing concerns of cross‐species transmission in light of the SARS‐CoV‐2 pandemic have made natural history reporting of co‐roosting, such has been naturally observed between pangolins and bats (Lehmann et al., 2020), even more important.

**TABLE 1:** Examples of co‐roosting multi‐species host systems, including pathogens assessed.

Roosting habitat	Co‐roosting species	Pathogen of concern mentioned or tested	Reference
Underground burrows	Giant pangolin (*Smutsia gigantea*); white‐bellied pangolin (*Phataginus tricuspis*); Old World leaf‐nosed bat sp. (*Hipposideridae* sp.); sac‐winged bat sp. (*Emballonuridae sp*); bent‐winged bat sp. (*Miniopterus* sp.); other unknown bat sp.	SARS‐CoV‐2	(Lehmann et al., 2020)
Chimney	Chimney swift (*Chaetura pelagica*); rock pigeon (*Columba livia*)		(Pearce & Foote, 2022)
Nest (brood parasitism)	Brown‐headed cowbirds (*Molothrus ate*r); common grackle (*Quiscalus quiscula*)		(Peer et al., 2001)
Nest box (nest predation)	House wren (*Troglodytes aedon*); tree swallow (*Tachycineta bicolor*)		(Finch, 1990)
Cave	Common vampire bat (*Desmodus rotundus*); Jamaican fruit bat (*Artibeus jamaicensis*); Seba's short‐tailed bat (*Carollia perspicillata*); hairy‐legged vampire bat (*Diphylla ecaudata*); little big‐eared bat (*Micronycteris megalotis*); cave myotis (*Myotis velifer*); Mexican funnel‐eared bat (*Natalus stramineus*); hairy‐legged myotis (*Myotis keaysi*); fringed myotis (*Myotis thysanodes*)		(Brunet & Medellín, 2001)
Building	Hairy‐legged vampire bat (*Diphylla ecaudata*); common vampire bat (*Desmodus rotundus*)	Rabies virus	(da Rosa et al., 2024)
Hollow tree cavity or underground tunnel	Common vampire bat (*Desmodus rotundus*); Pallas's long‐tongued bat (*Glossophaga soricina*); greater sac‐winged bat (*Saccopteryx bilineata*); fringe‐lipped bat (*Trachops cirrhosus*); big‐eared woolly bat (*Chrotopterus auritus*)	*Bartonella* spp.; hemosplasmas	(Becker et al., 2020, 2021)
Artificial roost in flight enclosure	Common vampire bat (*Desmodus rotundus*); pale spear‐nosed bat (*Phyllostomus discolor*); little yellow‐shouldered bat (*Sturnira lilum*)		(Wohlgenant, 1994)
Abandoned mines	Little brown bat (*Myotis lucifugus*); northern long‐eared bat (*Myotis septentrionalis*); tri‐colored bat (*Perimyotis subflavus*); big brown bat (*Eptesicus fuscus*)	*Pseudogymnoascus destructans*	(Hoyt et al., 2018)
Rocky outcrop den	Raccoon (*Procyon lotor*); Virginia opossum (*Didelphis virginiana*); striped skunk (*Mephitis mephitis*)		(Shirer & Fitch, 1970)
Tree cavity	Helmeted woodpecker (*Celeus galeatus*); white‐throated woodcreeper (*Xiphocolaptes albicollis*)		(Lammertink et al., 2019)
Underground burrow	Red fox (*Vulpes vulpes*); raccoon dog (*Nyctereutes procyonoides*); Japanese badger (*Meles anakuma*); Japanese weasel (*Mustela itatsi*); Japanese hare (*Lepus brachyurus*); unclassified rodent and bat species		(Kondo, 2018)
Underground den	Arctic fox (*Vulpes lagopus*); wolf (*Canis lupus*)		(Hendrickson et al., 2005)
Underground burrow	European badger (*Meles meles*); crested porcupine (*Hystrix cristata*); red fox (*Vulpes vulpes*); pine marten (*Martes martes*); stone marten (*Martes foina*); eastern cottontail (*Sylvilagus floridanus*); wood mouse (*Apodemus* sp.); brown rat (*Rattus norvegicus*); coypu (*Myocastor coypus*)		(Mori et al., 2015)
Sugarcane field	White‐tailed kite (*Elanus leucurus*); merlin (*Falco columbarius*); northern harriers (*Circus cyaneus*); American kestrel (*Falco sparverius*); white‐tailed hawk (*Buteo albicaudatus*); crested caracara (*Caracara cheriway*)		(Clark, 2006)

Theoretical models offer an important tool to reconcile evolutionary barriers to cross‐species transmission with the frequent contacts afforded by co‐roosting behavior. There is growing evidence for pathogen sharing within co‐roosting host systems (Becker et al., 2024; Bergner, Orton, et al., 2021) as well as pathogen sharing in these contexts being most common for closely related host species (McKee et al., 2019). However, phylogenetic contributions to pathogen sharing, and thus infection prevalence within the co‐roosting host community, can be challenging to estimate with limited empirical data. Despite prior calls for developing mathematical frameworks to study pathogen spread within multi‐host communities (Buhnerkempe et al., 2015; Lloyd‐Smith et al., 2009, 2015), we lack generalizable models that explicitly and flexibly consider phylogenetic barriers. Previous theory has considered how proportional reductions in transmission efficiency between two hosts affect pathogen spillover (Faust et al., 2018), and such work can be generalized and extended to consider the widely observed decline in cross‐species transmission with phylogenetic distance in multi‐host communities.

Here, we develop generalizable models for exploring cross‐species transmission in multi‐species, co‐roosting systems. We adapt common epidemiological modeling frameworks for pathogen transmission between two and three host species, incorporating greater transmission efficiency among closely related host species. Due to the nature of overlapping contact within and between co‐roosting host species, we focus our modeling framework for pathogens that are directly transmitted. Further, since infection‐induced mortality related to cross‐species transmission within wildlife co‐roosting systems is often unknown, our modeling framework is pertinent to pathogens with low virulence. To explore conditions for pathogen invasion and long‐term model behavior, we guided parameterization with Neotropical bat systems, which commonly display co‐roosting behavior between two or more species (Table 1) and harbor a wide range of zoonotic pathogens (Brunet & Medellín, 2001; Letko et al., 2002; Phelps et al., 2016). Our results support realistic contexts for cross‐species pathogen transmission in three ways. First, we show that pathogen invasion is a function of phylogenetic similarity among co‐roosting hosts, such that invasion can be limited by phylogenetic barriers under certain parameterizations. Second, infection prevalence across the co‐roosting community maximizes when host species are closely related, supporting positive relationships between cross‐species transmission and phylogenetic relatedness. Third, roost‐level infection prevalence also increases when hosts were distantly related, supporting increased pathogen spillover when hosts are regularly in close contact. Our modeling framework presented here can be modified and applied across many multi‐host systems where transmission requires close contact.

## METHODS

To determine how variable phylogenetic relatedness influences pathogen prevalence in a co‐roosting host community, we developed multiple sets of ordinary differential equation models describing population and infection dynamics. We coarsely parameterized these models around well‐studied bat systems, but our model structures are sufficiently generalizable to apply to the broad range of systems in which co‐roosting of host species and pathogen sharing can occur (Table 1).

### Infection dynamics of two co‐roosting hosts

We first consider the population dynamics of two host species occupying a shared roost environment, with two distinct population sizes (*N*_A_ and *N*_B_). Per‐capita reproduction for both species is described by *b*_0_ − *b*_1_ *N*, where *b*_0_ and *b*_1_ are the density‐independent and density‐dependent birth rates and *N* = *N*_A_ + *N*_B_, such that density dependence is driven by joint species occupancy. Both species likewise experience per‐capita mortality at rate μ, such that our model is most applicable to co‐roosting species with equivalent demographic rates. Similarly, this parameterization assumes negligible interspecific competition for resources within the roost, as observed for multiple woodpecker species in tree cavities (Lammertink et al., 2019) or multiple bat species within large caves or buildings (Brunet & Medellín, 2001, da Rosa et al., 2024; Table 1). The coupled population dynamics of the roost are thus defined by the following differential equations:

(1a) $$\frac{dN_{A}}{\mathrm{dt}}=\left(b_{0}-b_{1}N\right)N_{A}-\mu N_{A}$$

(1b) $$\frac{dN_{B}}{\mathrm{dt}}=\left(b_{0}-b_{1}N\right)N_{B}-\mu N_{B}$$

We next expand single‐species epidemiological models classifying hosts as susceptible (*S*), infected (*I*), and recovered (*R*) or latent (*L*), where the population size of each species is represented by *N*_*i*_ = *S*_*i*_ + *I*_*i*_ + *R*_*i*_|*L*_*i*_ (Anderson & May, 1979; Plowright et al., 2016). Pathogen transmission from infected to susceptible hosts within each species occurs at the rate β, and we assume equivalent intraspecific transmission rates among host species alongside density‐dependent contacts. Because the probability of cross‐species transmission typically declines for distantly related species across systems (Albery et al., 2021; Faria et al., 2013; Huang et al., 2014; Longdon et al., 2011; Parker et al., 2015; Pedersen & Davies, 2009; Streicker et al., 2012), we model interspecific transmission (θ) as an exponential function of host phylogenetic distance. Here, ψ represents proportional phylogenetic distance and λ is a shape parameter governing the strength of exponential decay on interspecific transmission (Appendix S2: Figure S1; Virgin et al., 2009). As our model considers distinct host species, interspecific transmission is always less than intraspecific transmission, and subscripts refer to transmission from the donor host species (*i*) to the recipient host species (*j*):

(2) $${ϴ}_{\mathrm{ij}}=\beta e^{\left(1-\psi_{\mathrm{ij}}\right)\lambda }$$

**FIGURE 1:**
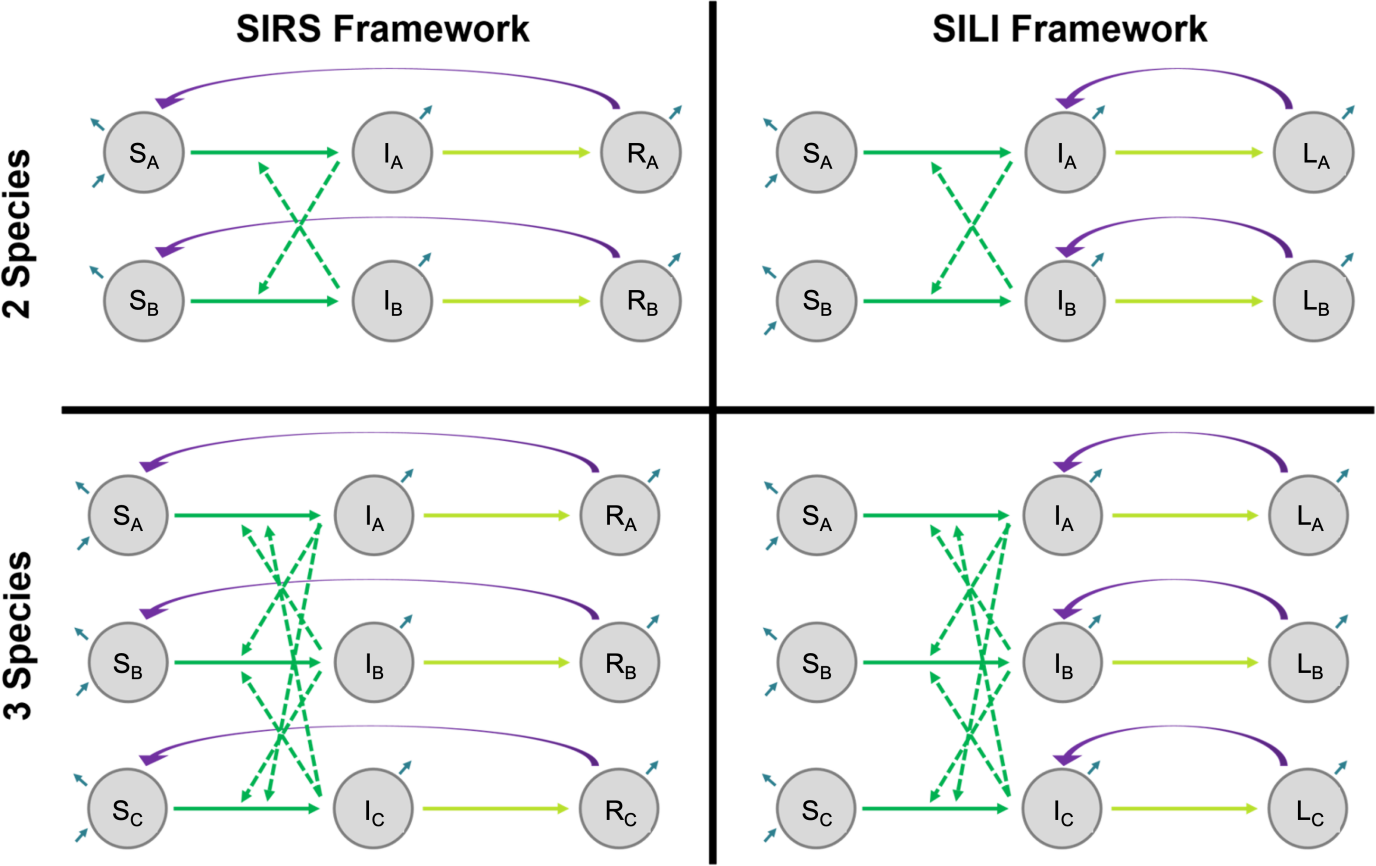
We created two‐ and three‐species compartmental models using SIRS and SILI frameworks. Colors represent processes for pathogen transmission (green), pathogen clearance (yellow), waning immunity or reactivation of latent infection (purple), and demography (blue).

Following transmission, infectious hosts recover or become latently infected at rate γ (i.e., 1/γ provides the infectious period). We thus consider a susceptible–infected–recovered–susceptible (SIRS) system and a susceptible–infected–latent–infected (SILI) system (Figure 1). Here, the rate ɛ governs the loss of protective immunity (SIRS model), whereas the rate ω governs the reactivation of latent (or chronic) infection (SILI model). Such frameworks are applicable to many pathogens that require close‐contact transmission, are temporarily immunizing, and exhibit general chronic infection or true latency (Virgin et al., 2009). We do not consider disease‐induced mortality, such that our models are most applicable to low‐virulence pathogens (e.g., those with strict coevolutionary history with a particular host clade). We therefore describe SIRS dynamics by the following equations:

(3a) $$\frac{dS_{A}}{\mathrm{dt}}=\left(b_{0}-b_{1}\;N\right)N_{A}-\left(\;{\beta S}_{A}\;I_{A}+ϴS_{A}\;I_{B}\right)-\mu S_{A}+\epsilon R_{A}$$

(3b) $$\frac{dI_{A}}{\mathrm{dt}}=\left(\beta S_{A}\;I_{A}+ϴS_{A}\;I_{B}\right)-\left(\mu +\gamma \right)I_{A}$$

(3c) $$\frac{dR_{A}}{\mathrm{dt}}=\gamma I_{A}-\left(\mu +\epsilon \right)R_{A}$$

(3d) $$\frac{dS_{B}}{\mathrm{dt}}=\left(b_{0}-b_{1}\;N\right)N_{B}-\left(\;{\beta S}_{B}\;I_{B}+ϴS_{B}\;I_{A}\right)-\mu S_{B}+\epsilon R_{B}$$

(3e) $$\frac{dI_{B}}{\mathrm{dt}}=\left(\beta S_{B}\;I_{B}+ϴS_{B}\;I_{A}\right)-\left(\mu +\gamma \right)I_{B}$$

(3f) $$\frac{dR_{B}}{\mathrm{dt}}=\gamma I_{B}-\left(\mu +\epsilon \right)R_{B}$$

Similarly, SILI dynamics are given by the following equations:

(4a) $$\frac{dS_{A}}{\mathrm{dt}}=\left(b_{0}-b_{1}\;N\right)N_{A}-\left(\beta S_{A}\;I_{A}+ϴS_{A}\;I_{B}\right)-\mu S_{A}$$

(4b) $$\frac{dI_{A}}{\mathrm{dt}}=\left(\beta S_{A}\;I_{A}+ϴS_{A}\;I_{B}\right)-\left(\mu +\gamma \right)I_{A}+\omega L_{A}$$

(4c) $$\frac{dL_{A}}{\mathrm{dt}}=\gamma I_{A}-\left(\mu +\omega \right)L_{A}$$

(4d) $$\frac{dS_{B}}{\mathrm{dt}}=\left(b_{0}-b_{1}\;N\right)N_{B}-\left(\beta S_{B}\;I_{B}+ϴS_{B}\;I_{A}\right)-\mu S_{B}$$

(4e) $$\frac{dI_{B}}{\mathrm{dt}}=\left(\beta S_{B}\;I_{B}+ϴS_{B}\;I_{A}\right)-\left(\mu +\gamma \right)I_{B}+\omega L_{B}$$

(4f) $$\frac{dL_{B}}{\mathrm{dt}}=\gamma I_{B}-\left(\mu +\omega \right)L_{B}$$

### Infection dynamics of three co‐roosting hosts

We next expand Equations (3) and (4) to consider three host species sharing a single roost. We again first consider population dynamics, now of three host species (*N*_A_, *N*_B_ and *N*_C_), such that *N* = *N*_A_ + *N*_B_ + *N*_C_. Similar to two‐species models, demographic rates were the same for each species, but the strength of density dependence in birth is now shaped by occupancy of all three species. We therefore describe SIRS dynamics in the three‐species co‐roosting system by the following equations:

(5a) $$\frac{dS_{A}}{\mathrm{dt}}=\left(b_{0}-b_{1}N\right)N_{A}-\left(\beta S_{A}\;I_{A}+{ϴ}_{\mathrm{AB}}\;S_{A}\;I_{B}+{ϴ}_{\mathrm{AC}}\;S_{A}\;I_{C}\right)-\mu S_{A}+\epsilon R_{A}$$

(5b) $$\frac{dI_{A}}{\mathrm{dt}}=\left(\beta S_{A}\;I_{A}+{ϴ}_{\mathrm{AB}}\;S_{A}\;I_{B}+{ϴ}_{\mathrm{AC}}\;S_{A}\;I_{C}\right)-\left(\mu +\gamma \right)I_{A}$$

(5c) $$\frac{dR_{A}}{\mathrm{dt}}=\gamma I_{A}-\left(\mu +\epsilon \right)R_{A}$$

(5d) $$\frac{dS_{B}}{\mathrm{dt}}=\left(b_{0}-b_{1}\;N\right)N_{B}-\left(\beta S_{B}\;I_{B}+{ϴ}_{\mathrm{BA}}\;S_{B}\;I_{A}+{ϴ}_{\mathrm{BC}}\;S_{B}\;I_{C}\right)-\mu S_{B}+\epsilon R_{B}$$

(5e) $$\frac{dI_{B}}{\mathrm{dt}}=\left(\beta S_{B}\;I_{B}+{ϴ}_{\mathrm{BA}}\;S_{B}\;I_{A}+{ϴ}_{\mathrm{BC}}\;S_{B}\;I_{C}\right)-\left(\mu +\gamma \right)I_{B}$$

(5f) $$\frac{dR_{B}}{\mathrm{dt}}=\gamma I_{B}-\left(\mu +\epsilon \right)R_{B}$$

(5g) $$\frac{dS_{C}}{\mathrm{dt}}=\left(b_{0}-b_{1}\;N\right)N_{C}-\left(\beta S_{C}\;I_{C}+{ϴ}_{\mathrm{CA}}\;S_{C}\;I_{A}+{ϴ}_{\mathrm{CB}}\;S_{C}\;I_{B}\right)-\mu S_{C}+\epsilon R_{C}$$

(5h) $$\frac{dI_{C}}{\mathrm{dt}}=\left(\beta S_{C}\;I_{C}+{ϴ}_{\mathrm{CA}}\;S_{C}\;I_{A}+{ϴ}_{\mathrm{CB}}\;S_{C}\;I_{B}\right)-\left(\mu +\gamma \right)I_{C}$$

(5i) $$\frac{dR_{C}}{\mathrm{dt}}=\gamma I_{C}-\left(\mu +\epsilon \right)R_{C}$$

Similarly, SILI dynamics under three co‐roosting species is given by the following equations:

(6a) $$\frac{dS_{A}}{\mathrm{dt}}=\left(b_{0}-b_{1}\;N\right)N_{A}-\left(\beta S_{A}\;I_{A}+{ϴ}_{\mathrm{AB}}\;S_{A}\;I_{B}+{ϴ}_{\mathrm{AC}}\;S_{A}\;I_{C}\right)-\mu S_{A}$$

(6b) $$\frac{dI_{A}}{\mathrm{dt}}=\left(\beta S_{A}\;I_{A}+{ϴ}_{\mathrm{AB}}\;S_{A}\;I_{B}+{ϴ}_{\mathrm{AC}}\;S_{A}\;I_{C}\right)-\left(\mu +\gamma \right)I_{A}+\omega L_{A}$$

(6c) $$\frac{dL_{A}}{\mathrm{dt}}=\gamma I_{A}-\left(\mu +\omega \right)L_{A}$$

(6d) $$\frac{dS_{B}}{\mathrm{dt}}=\left(b_{0}-b_{1}\;N\right)N_{B}-\left(\beta S_{B}\;I_{B}+{ϴ}_{\mathrm{BA}}\;S_{B}\;I_{A}+{ϴ}_{\mathrm{BC}}\;S_{B}\;I_{C}\right)-\mu S_{B}$$

(6e) $$\frac{dI_{B}}{\mathrm{dt}}=\left(\beta S_{B}\;I_{B1}+{ϴ}_{\mathrm{BA}}\;S_{B}\;I_{A}+{ϴ}_{\mathrm{BC}}\;S_{B}\;I_{C}\right)-\left(\mu +\gamma \right)I_{B}+\omega L_{B}$$

(6f) $$\frac{dL_{B}}{\mathrm{dt}}=\gamma I_{B}-\left(\mu +\omega \right)L_{B}$$

(6g) $$\frac{dS_{C}}{\mathrm{dt}}=\left(b_{0}-b_{1}\;N\right)N_{C}-\left(\beta S_{C}\;I_{C}+{ϴ}_{\mathrm{CA}}\;S_{C}\;I_{A}+{ϴ}_{\mathrm{CB}}\;S_{C}\;I_{B}\right)-\mu S_{C}$$

(6h) $$\frac{dI_{C}}{\mathrm{dt}}=\left(\beta S_{C}\;I_{C}+{ϴ}_{\mathrm{CA}}\;S_{C}\;I_{A}+{ϴ}_{\mathrm{CB}}\;S_{C}\;I_{B1}\right)-\left(\mu +\gamma \right)I_{C}+\omega L_{C}$$

(6i) $$\frac{dL_{C}}{\mathrm{dt}}=\gamma I_{C}-\left(\mu +\omega \right)L_{C}$$

### Model parameterization

We parameterized our models around co‐roosting bat species in Central and South America, as Neotropical bats are known to spatially partition within shared roosts (Wohlgenant, 1994), limiting interspecific competition. We considered common vampire bats (Phyllostomidae: *Desmodus rotundus*) as the focal host species, as they commonly co‐roost with different bat species that span closely and distantly related families (Table 1). For example, in Costa Rica, vampire bats roost in caves with bat species from at least three families (e.g., Vespertilionidae: hairy‐legged myotis [*Myotis keaysi*], fringed myotis [*Myotis thysanodes*], cave myotis [*Myotis velifer*]; Natalidae: Mexican funnel‐eared bat [*Natalus stramineus*]; Phyllostomidae: Jamaican fruit bat [*Artibeus jamaicensis*], Seba's short‐tailed bat [*Carollia perspicillata*], little big‐eared bat [*Micronycteris megalotis*]) and even other vampire bat species (Table 1; Brunet & Medellín, 2001). In Belize, common vampire bats roost in hollow trees or abandoned temples with bat species from at least two families (Phyllostomidae: Pallas's long‐tongued bat [*Glossophaga soricina*], fringe‐lipped bat [*Trachops cirrhosus*], big‐eared woolly bat [*Chrotopterus auritus*]; Emballonuridae: greater sac‐winged bat [*Saccopteryx bilineata*] Rate; Table 1; Becker et al., 2020, 2021). As a highly social species, vampire bats have fine‐scale connectivity networks within their colonies (Ripperger et al., 2019) and also share pathogens between co‐roosting bat species as well as other non‐bat wildlife, livestock, and humans due to their unique diet of blood (Becker et al., 2020, 2021; Condori‐Condori et al., 2013; Lord et al., 1975; Schneider et al., 2009; Teider‐Junior et al., 2021). Within the context of this system, we parameterized infection processes around coronaviruses and herpesviruses, which circulate in diverse Neotropical bat species (including common vampire bats) and exhibit SIRS and SILI dynamics, respectively (Bergner, Mollentze, et al., 2021; Corman et al., 2013; Griffiths et al., 2023; Moreira Marrero et al., 2021).

We similarly parameterized host demographics around Neotropical bat systems. We chose a maximum per capita birth rate (*b*_0_) to be two births each year, which is the maximum annual fecundity of most Neotropical bat species (Table 2; Wilkinson & South, 2002). We also assumed an equal starting population size among all host species (*N*_A_ *= N*_B_, *N*_A_ *= N*_B_ *= N*_C_). Under this assumption, we initialized our models with each species starting with 1000 bats, representing a large colony of many Neotropical bat species (Santana et al., 2011). The total population in three‐species models when all population sizes were equal (3000 bats) also denoted our carrying capacity (*K*) (Table 2).

**TABLE 2:** Descriptions of parameters and parameter space used in two‐ and three‐species SIRS and SILI models. All units are in days unless otherwise noted.

Process	Parameter	Description	Parameter space	Citation
Transmission	β	Intraspecific transmission rate	0.0001, 0.0005, 0.001	(Jeong et al., 2017)
	ϴ	Interspecific transmission rate	β*e* ^(1−Ψ)λ^	Appendix S2: Figure S1 (Albery et al., 2021, Otto & Day, 2007)
	λ	Exponential decay curve shape parameter	−1, −5, −10	Appendix S2: Figure S1
	Ψ	Phylogenetic relatedness	0.0001–0.9999	Figure 2 (Upham et al., 2019)
Pathogen clearance	γ	Infection clearance rate	Short: 1/5 Long: 1/548	(Griffiths et al., 2023, Jeong et al., 2017)
Waning immunity (SIRS model only)	ε	Rate of immunity loss	Short: 1/60 Long: 1/1643	(Crellen et al., 2021, Griffiths et al., 2023, Jeong et al., 2017)
Reactivation (SILI model only)	ω	Reactivation rate	Short: 1/60 Long: 1/1643	(Crellen et al., 2021, Griffiths et al., 2023, Jeong et al., 2017)
Demography	μ	Natural death rate of all species	1/(12*365)	(De Magalhães & Costa, 2009)
	*b*_0_	Maximum per capita birth rate	2 bats/365	(Wilkinson & South, 2002)
	*b*_1_	Density‐dependent per capita birth rate	(*b*_0_ − μ)/*K*	
	*K*	Carrying capacity	3000	
	*N*_A_, *N*_B_, *N*_C_	Population size of species A (*S*_A_ + *I*_A_ + *R*_A_), B (*S*_B_ + *I*_B_ + *R*_B_), or C (*S*_C_ + *I*_C_ + *R*_C_)	Starting size: 1000	(Dalquest, 1955, Streicker et al., 2012, Wimsatt, 1969)
	*N*	Total population size	*N*_A_ + *N*_B_ OR *N*_A_ + *N*_B_ + *N*_C_	

We set natural mortality rates (μ) in our two‐ and three‐species models based on average Neotropical bat lifespans. For example, common vampire bats live around 6 years in the wild, while other known co‐roosting bat species can live a maximum of 6 to 20 years in the wild (e.g., sac‐winged bat, *Saccopteryx bilineata*: 6 years maximum wild lifespan; hairy‐legged vampire bat, *Diphylla ecaudata*: 8 years maximum wild lifespan; pale spear‐nosed bat, *Phyllostomus discolor*: 9 years maximum wild lifespan; cave myotis, *Myotis velifer*: 11.3 years maximum wild lifespan; fringed myotis, *Myotis thysanodes*: 18.3 years maximum wild lifespan). Therefore, we parameterized mortality rates equally across all hosts on a 12‐year lifespan.

Lastly, we coarsely parameterized pathogen traits based on prior studies of bat–virus interactions. For intraspecific transmission rates (β), we used estimates for alphacoronavirus infection in Australian southern myotis (*Myotis macropus*; Table 2; Jeong et al., 2017). We also systematically explored lower intraspecific transmission rates to consider cases of less frequent contact between hosts of the same species. Pathogen clearance rates (γ) and rates of waning immunity (𝜖; SIRS models) or infection reactivation (ω; SILI models) were parameterized under short or long conditions (Table 2), since these processes remain poorly understood in bats (Gentles et al., 2020; Glennon et al., 2019), but we always paired short or long rates together. Short parameterizations represent coronavirus dynamics and were chosen to fall within previously published rates for transient alphacoronavirus infections in Australian southern myotis (Table 2; Jeong et al., 2017). Long parameterizations represent herpesvirus dynamics and were selected from maximum likelihood estimates of clearance and reactivation rates in vampire bats (Table 2; Griffiths et al., 2023). Finally, we factorally manipulated the effect of phylogenetic distance on interspecific transmission (λ), representing variation in coevolutionary relationships between pathogens and their hosts (e.g., pathogen growth, replication, and recombination rates; Shaw et al., 2020).

### Modeling approach

**FIGURE 2:**
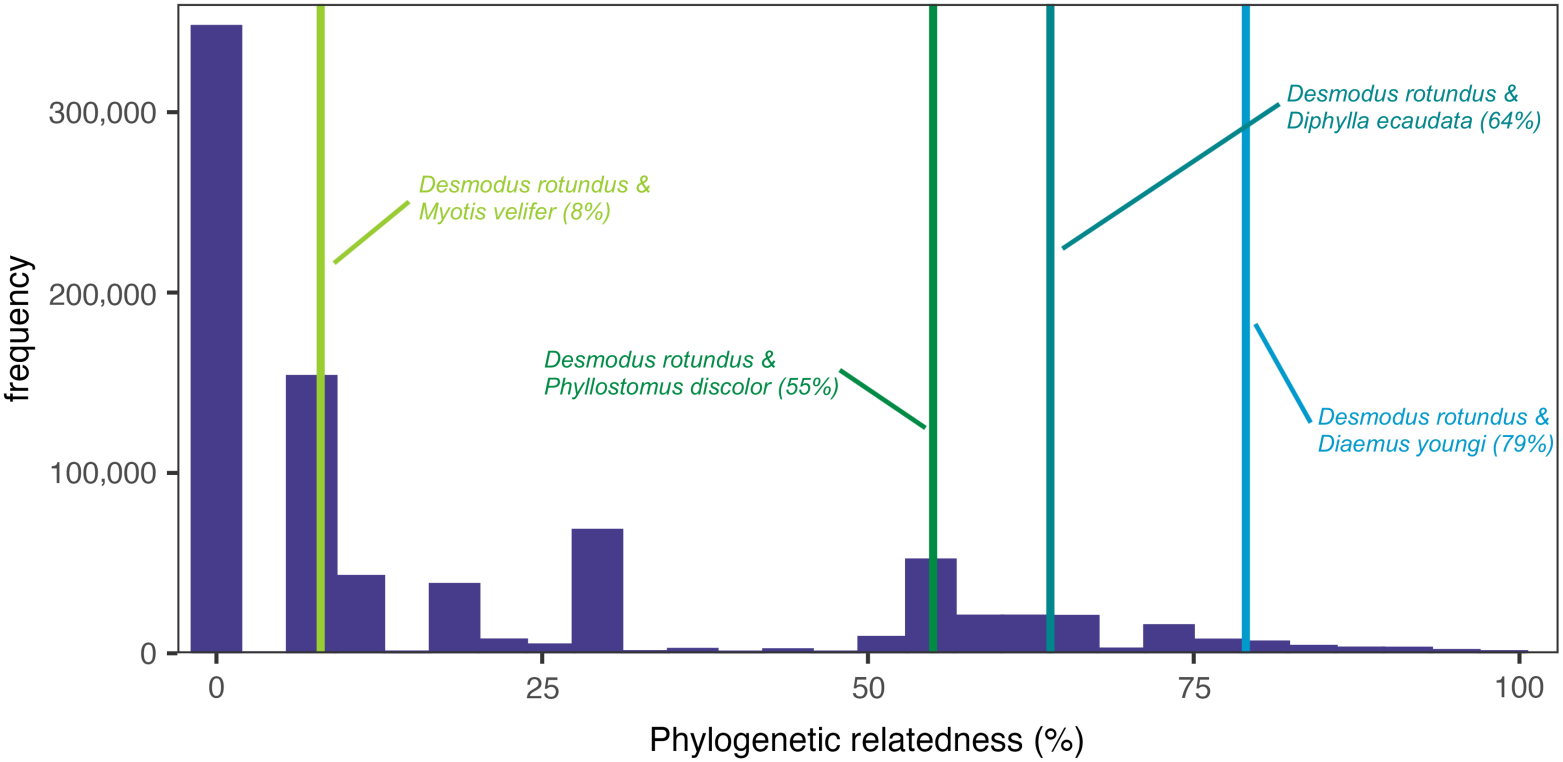
Distribution of phylogenetic relatedness among bats using the most recent mammalian phylogeny (Upham et al., 2019). Vertical lines represent specific examples of phylogenetic relatedness between *Desmodus rotundus* and other Neotropical bat species, as we tested model sensitivity by focusing on parameterization for *D. rotundus* and potential co‐roosting bat species.

To understand the role of host evolutionary history to infection dynamics in the context of co‐roosting, we first used the next‐generation matrix to derive the basic reproductive number (*R*_0_), a threshold quantity governing pathogen invasion and outbreak severity (Diekmann et al., 2010), for both SIRS and SILI models in their two‐ and three‐host systems (Appendix S1). We next determined how phylogenetic relatedness of co‐roosting hosts affected equilibrium infection prevalence (two species: (*I*_A_ + *I*_B_)/*N*; three species: (*I*_A_ + *I*_B_ + *I*_C_)/*N*) by numerically solving the model in the statistical environment R version 4.3.1 (R Core Team, 2023) using the *deSolve* package (Soetaert, 2010). We systematically covaried phylogenetic relatedness between paired hosts (ѱ; Figure 2), the effect of phylogenetic distance on interspecific transmission (λ), and the intraspecific transmission rate (β) for both SIRS and SILI frameworks, first for two‐species models and then for three‐species models. We also considered the sensitivity of our results to pathogen traits. Specifically, we simulated models across all factorial combinations of infection durations (1/γ) and durations of temporary immunity (1/𝜖; SIRS models) or latency (1/ω; SILI models). We paired short infectious periods with short durations of immunity or latency (i.e., coronaviruses) and long infectious periods with long durations of immunity or latency (i.e., herpesviruses). As such, each two‐ and three‐species model was simulated across 378 and 166,698 unique parameter combinations, respectively.

We simulated each model until equilibrium, using a simulation length of 300 years to ensure that the total and infectious population of the roost reached steady state (i.e., a difference of ≤ 0.001 individuals between the final 60 days of the time series; Appendix S2: Figures S2–S5). Simulations for three‐species SIRS and SILI models were performed remotely through the University of Oklahoma's Supercomputing Center for Education & Research. Results were visualized using the *ggplot2* and *ggtern* packages in R version 4.3.1 (Hamilton & Ferry, 2018; R Core Team, 2023; Wickham, 2016).

## RESULTS

### Effects of co‐roosting on pathogen invasion

We first derived the conditions for pathogen invasion (*R*_0_ > 1) using the next‐generation matrix across our two‐ and three‐species co‐roosting SIRS and SILI models (Appendix S1). For the two‐host SIRS model, *R*_0_ is an intuitive product of the disease‐free equilibrium population size (i.e., *K/2* for each host species under our parameterization of equivalent starting conditions and demographic rates), intra‐ and inter‐specific transmission (the latter a function of phylogenetic similarity), and the infectious period:

$$R_{0}=\frac{K\beta \left(1+e^{\left(1-\psi \right)\lambda }\right)}{2\left(\mu +\gamma \right)}$$

For the two‐host SILI model, the expression for *R*_0_ includes added complexity owing to the contribution of not only infectious but also latently infected individuals to infection dynamics:

$$R_{0}=\frac{K\beta \left(1+e^{\left(1-\psi \right)\lambda }\right)\left(\mu +\omega \right)}{2\mu \left(\mu +\gamma +\omega \right)}$$

Within both derivations of *R*_0_, pathogen invasion is a positive, monotonic function of phylogenetic relatedness. Under shared parameterizations, however, pathogen invasion success within these two‐species co‐roosting systems is substantially greater for infections with cycles of latency and reactivation (i.e., SILI processes) than for those with waning immunity (i.e., SIRS processes), owing to the additional contributions of pathogen relapse to the infectious period.

For three‐host models, we derived more complex expressions for pathogen invasion, resulting from cubic solutions to the dominant eigenvalue of each next‐generation matrix. For the SIRS model, we first define *P*, the sum of the reciprocal transmission among host species pairs, and *C*, the sum of directional transmission cycles among the three host species:

$$P=\frac{\theta_{\mathrm{AB}}^{2}+\theta_{\mathrm{BC}}^{2}+\theta_{\mathrm{AC}}^{2}}{\beta^{2}}$$

$$C=\frac{2\left(\theta_{\mathrm{AB}}\theta_{\mathrm{AC}}\theta_{\mathrm{BC}}\right)}{\beta^{3}}$$

Using the cubic solution, we then derive Δ as the discriminant and Λ as the largest root:

$$\Delta ={\left(\frac{C}{2}\right)}^{2}-{\left(\frac{P}{3}\right)}^{3}$$

$$\Lambda =1+\sqrt[{3}]{\frac{C}{2}+\sqrt{\Delta }}+\sqrt[{3}]{\frac{C}{2}-\sqrt{\Delta }}$$

With these terms, we can then define *R*_0_, which here is a product of the disease‐free equilibrium population size, intra‐specific transmission, infectious period, and the contributions of both two‐ and three‐species cross‐species transmission terms:

$$R_{0}=\frac{K\beta }{3\left(\mu +\gamma \right)}\Lambda$$

We can leverage these expressions for *P*, *C*, Δ, and Λ to similarly derive *R*_0_ for the SILI model, which, as with its two‐host counterpart, includes added complexity due to the contributions of not only infectious but also latently infected individuals to infection dynamics:

$$R_{0}=\frac{K\beta \left(\mu +\omega \right)}{3\mu \left(\mu +\gamma +\omega \right)}\Lambda$$

**FIGURE 3:**
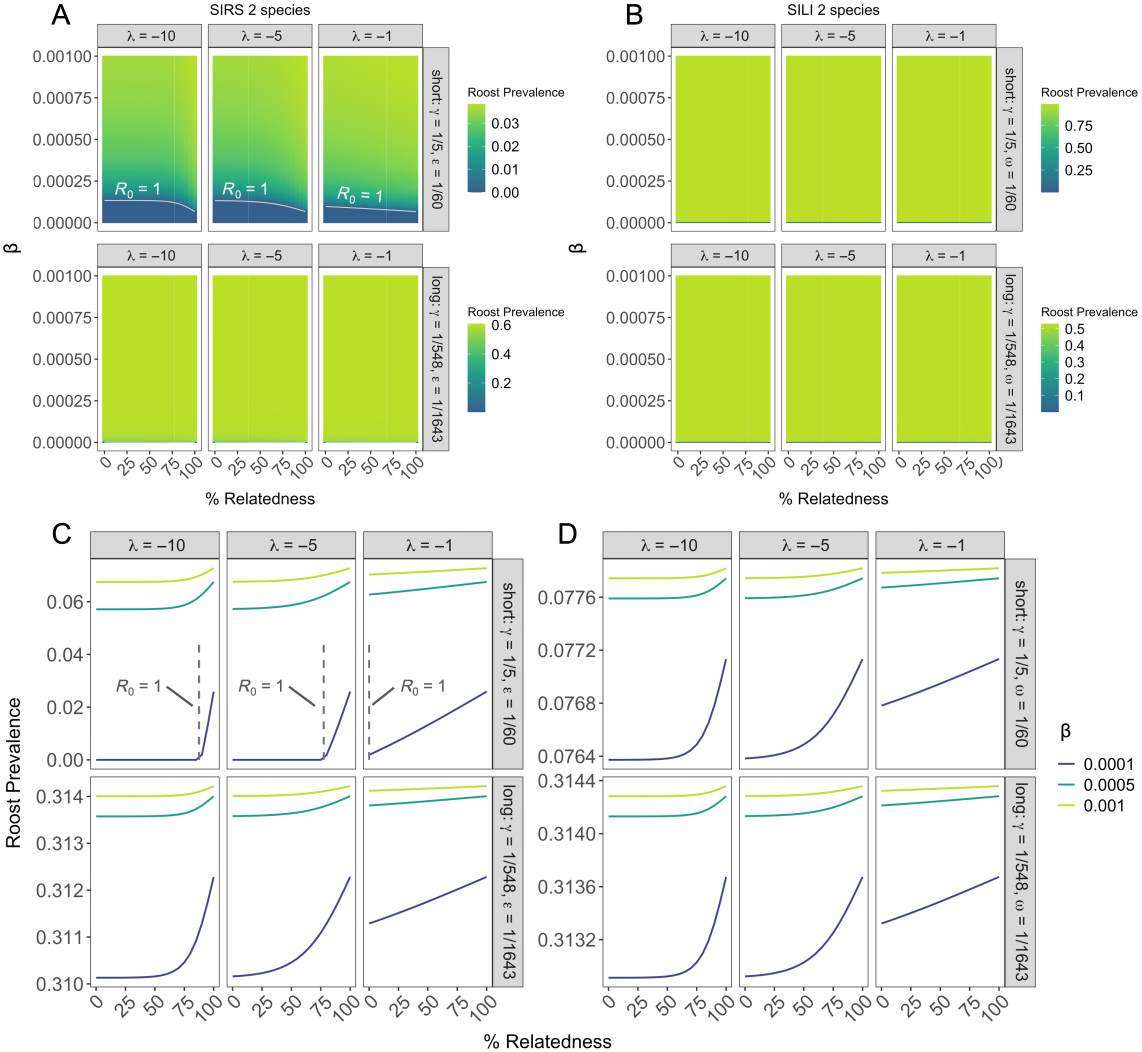
Pathogen invasion thresholds (A, B) and roost prevalence at equilibrium (A–D) for two‐species SIRS (A, C) and SILI models (B, D) under varying intraspecific transmission (β), effects of phylogenetic relatedness on interspecific transmission (λ), infectious periods (1/γ), and immune (1/ε; SIRS only) or latent periods (1/ω; SILI only). Under our parameterizations, *R*_0_ is greater than one in all contexts (B–D) except for two‐species SIRS models with short periods of infection and immunity (A). Roost prevalence increases with phylogenetic relatedness and intraspecific transmission, and stronger effects of phylogenetic relatedness on interspecific transmission (i.e., decreasing λ) limit increases to roost prevalence. SIRS and SILI models yield similar patterns. The gray dotted vertical line segment is representative of *R*_0_ = 1 when β = 0.0001 in short periods for SIRS models only. All other prevalence values are associated with *R*_0_ > 1. Note that roost prevalence scales are different across panels.

**FIGURE 4:**
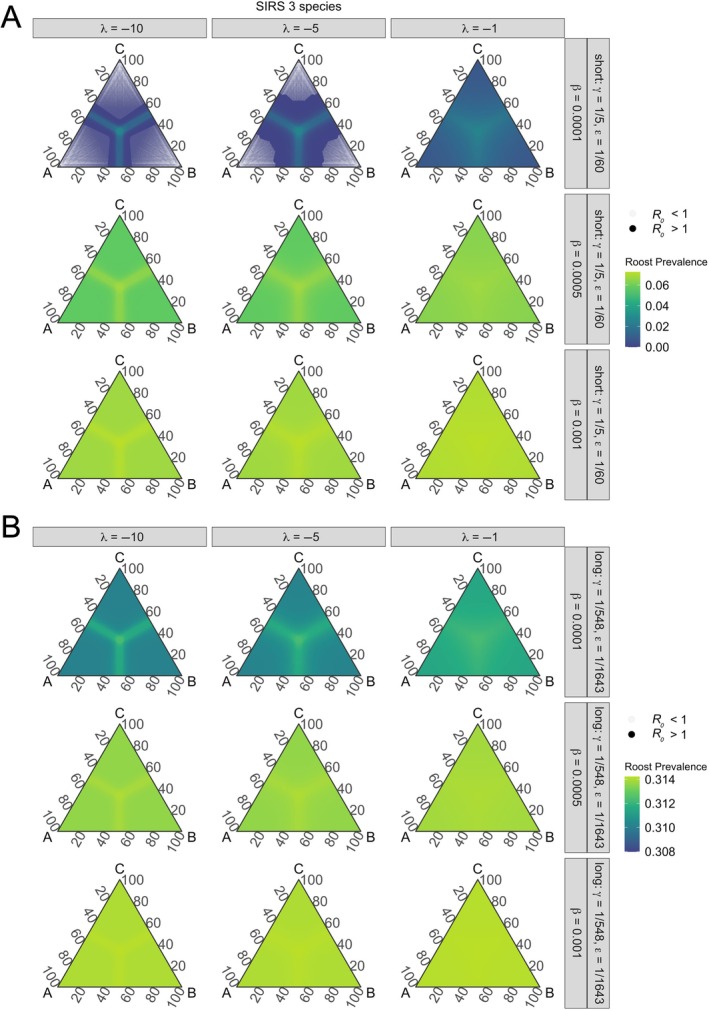
Pathogen invasion thresholds and roost prevalence at equilibrium for three‐species SIRS models under varying intraspecific transmission (β), effects of phylogenetic distance on interspecific transmission (λ), and short (A) or long (B) infectious periods (1/γ) and immunity periods(1/ε; SIRS only). Within our parameterizations, *R*_0_ is greater than one in most contexts except for when transmission is lowest and the effects of phylogenetic relatedness are moderate and greatest in SIRS models under short infections and immunity periods (A). Overall, roost prevalence is variable with phylogenetic relatedness. Edges of ternary plots represent percent relatedness between two species, and species are labeled at each vertex. Species are more closely and evenly related moving toward the center of ternary plots, and thus become more distantly and unevenly related moving toward the plot edges. Translucent points represent areas of phylogenetic space where *R*_0_ < 1. Note that roost prevalence scales are different across panels.

**FIGURE 5:**
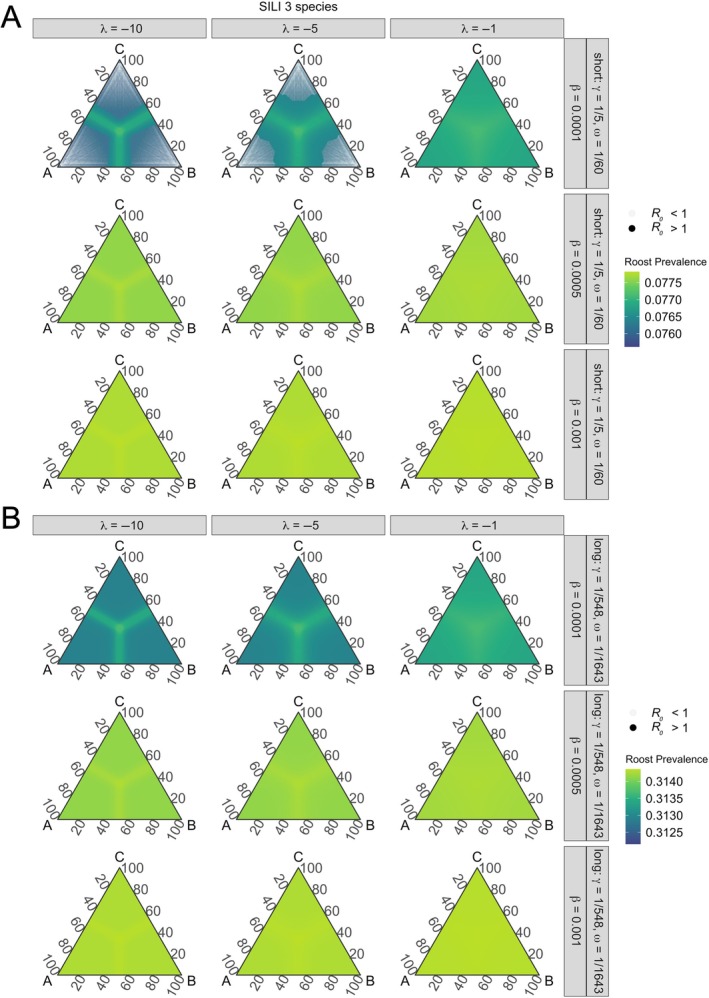
Pathogen invasion thresholds and roost prevalence at equilibrium for three‐species SILI models under varying intraspecific transmission (β), effects of phylogenetic distance on interspecific transmission (λ), and short (A) or long (B) infectious periods (1/γ) and latent periods (1/ω; SILI only). Within our parameterizations, *R*_0_ is greater than one in most contexts except for when transmission is lowest and the effects of phylogenetic relatedness are moderate and greatest in SILI models under short infections and latency periods (A). Overall, roost prevalence is variable with phylogenetic relatedness. Edges of ternary plots represent percent relatedness between two species, and species are labeled at each vertex. Species are more closely and evenly related moving toward the center of ternary plots, and thus become more distantly and unevenly related moving toward the plot edges. Translucent points represent areas of phylogenetic space where *R*_0_ < 1. Note that roost prevalence scales are different across panels.

In the context of Neotropical bat systems, pathogen invasion (*R*_0_ > 1) occurred under all factorial parameterizations of two‐ and three‐species SIRS and SILI models (Figures 3, 4, and 5). However, we identified specific parameterizations where phylogenetic distance limited pathogen invasion within co‐roosting host communities. In two‐species SIRS models, pathogen invasion could not occur across phylogenetic space at extremely low intraspecific transmission (β = 0.0001) and with short infectious and immunity periods (Figure 3A). However, those evolutionary restrictions to cross‐species transmission were modified by the strength of phylogenetic distance effects (λ; Figure 3A). We also identified contexts in which phylogenetic similarity limited pathogen invasion for our three‐species SIRS and SILI models (Figures 4 and 5). These models identified phylogenetic thresholds for pathogen invasion under the lowest intraspecific transmission rate (β = 0.0001), moderate and greatest strength of phylogenetic distance (λ = −5, −10), and with short infectious and immunity or latency periods (Figures 4A and 5A).

### Effects of co‐roosting on equilibrium infection prevalence

After deriving the conditions for pathogen invasion, we numerically solved our models to assess impacts of evolutionary history on equilibrium roost‐level infection prevalence (hereafter “roost prevalence”). Two‐ and three‐species SIRS and SILI models generally yielded qualitatively similar patterns, but SILI models yielded slightly greater roost prevalence than SIRS models. For all SILI and SIRS models, differences in model equilibria were minimal across parameter space, with roost prevalence varying by less than 2% with phylogenetic relatedness under short or long periods of infection and immunity or latency. Roost prevalence was maximized under increasing intraspecific transmission, longer infectious periods, and shorter durations of immunity or latency (Figures 3, 4, 5). When considering effects of phylogenetic relatedness (λ), we identified inflections of roost prevalence as a function of host similarity (Figures 3, 4, 5). In two‐ and three‐species models, roost prevalence increased when co‐roosting species were more than approximately 50% or more related, with cross‐species transmission thresholds relaxing as phylogenetic distance had weaker effects on interspecific transmission (i.e., with increasing λ; Figures 3, 4, and 5). Further, in three‐species models, while roost prevalence was maximized when all species were closely related (i.e., the center of ternary plots; Figure 3), increased roost prevalence was also expanded across phylogenetic space along an approximate 50% threshold for all species as they became more distantly related (Figures 4 and 5).

## DISCUSSION

Co‐roosting between multiple host species can facilitate cross‐species transmission due to frequent interactions between hosts (Reluga et al., 2007). Here, we created a generalizable model framework for understanding how phylogenetic relatedness influences host pathogen prevalence in the context of this co‐roosting environment, considering pathogens requiring close contact for transmission. Our derivations of *R*_0_ demonstrate a key role of phylogenetic similarity in pathogen invasion, with epidemic success increasing for more closely related host communities. Within our parameterization around Neotropical bat hosts, phylogenetic‐dependent transitions between the disease‐free equilibrium and pathogen invasion were primarily observed under very low intraspecific transmission rates and short periods of infection and immunity or latency. Such results indicate the potential for co‐roosting to facilitate increasing spillover risk for otherwise poorly transmissible infections. When numerically solving our two‐ and three‐species models, we found that roost prevalence at equilibrium generally was greatest when hosts were more closely related. However, we also identified contexts where equilibrium prevalence was maximized when species were more distantly related, signifying that multi‐host communities may not always follow strict relationships for increasing prevalence with phylogenetic relatedness (Albery et al., 2021; Faria et al., 2013; Huang et al., 2014; Longdon et al., 2011; Parker et al., 2015; Pedersen & Davies, 2009; Streicker et al., 2012).

Phylogenetic relatedness between host species has long been observed to be a strong driver of cross‐species transmission (Albery et al., 2021, Faria et al., 2013, Huang et al., 2014, Longdon et al., 2011, Parker et al., 2015, Pedersen & Davies, 2009, Streicker et al., 2012). While roost prevalence changes across phylogenetic space were relatively minimal, our SIRS and SILI models provide further theoretical support for these findings. However, we also found specific contexts in which roost prevalence departed from this assumption. In all three‐species SIRS and SILI models, we found increased roost prevalence when species were distantly and unevenly related. While these results are seemingly contradictory to our understanding of phylogenetic barriers to cross‐species transmission, such risks also increase with overlapping geography and decrease with host species dispersal (Albery et al., 2021; Luis et al., 2015; Pedersen & Davies, 2009; Streicker et al., 2012). Further, frequent interactions between susceptible and infected hosts can facilitate interspecific transmission (Reluga et al., 2007). For example, geographic overlap among host species more frequently resulted in coronavirus sharing at global scales than phylogenetic relationships (Leopardi et al., 2018). In our models, we considered a single community of co‐roosting host species and did not account for immigration, emigration, or competition between hosts, representing co‐roosting as a strong overlap in space with shared effects on population growth. Therefore, contexts in which roost prevalence was highest between species more distantly and unevenly related suggest that extreme spatial overlap in host communities (i.e., co‐roosting) may sometimes disrupt normal coevolutionary patterns between host species and their pathogens, even at relatively low levels of intraspecific transmission.

Gregariousness has previously been shown to be a strong predictor of cross‐species transmission at macroecological scales across taxa and in bats specifically, which is relevant given our explicit model parameterization (Altizer et al., 2003; Luis et al., 2015; Patterson & Ruckstuhl, 2013). Past theory in single‐host systems suggests that infection prevalence quickly saturates following an epidemic for large communal roosts (Laughlin et al., 2019). Here, starting populations of co‐roosting species and our selected carrying capacity represent a large roost community. Yet, distinct from prior single‐host theory, both two‐ and three‐species models at our lowest intraspecific transmission rates still identified phylogenetic barriers for pathogen invasion and thereby cross‐species transmission, manifesting in greater roost prevalence as host similarity increased. Even though these increases in roost prevalence across phylogenetic space were overall minor (i.e., 2% or less), thresholds of phylogenetic relatedness were maintained across short and long durations of infection and immunity or latency as well as stronger effects of phylogenetic distance on interspecific transmission. Therefore, phylogenetic relatedness of co‐roosting hosts in highly gregarious populations may drive the ability of pathogens to invade roost communities and facilitate sharp increases in roost prevalence over long timescales, even when intra‐ and interspecific transmission rates are low. While our population size parameterizations are applicable to highly gregarious co‐roosting systems (e.g., cave bats in Mexico with greater than 1000 individuals; Arita, 1993; Table 1), future applications of our model to smaller co‐roosting populations could find varying effects of phylogenetic distance depending on the magnitude of demographic differences across host species.

While the co‐roosting infection dynamics from the models and parameterization presented here change roost prevalence by only 2% or less, these relatively small changes highlight the risk of continued outbreaks and epidemics, particularly for SIRS infection contexts. Thus, the resulting number of infectious hosts in large, co‐roosting populations could still have important contributions to the force of infection and cross‐species transmission risk. Additionally, given that many wildlife species are highly mobile, particularly bats as parameterized here via flight, an additional 2% of infectious individuals could disperse pathogens over large spatial scales to infect additional species not accounted for within the co‐roosting system. Explicitly tracking the force of infection generated from co‐roosting donor species in our models (e.g., bats) to recipient hosts within a typical foraging radius (e.g., livestock and humans) could directly predict spillover risks (Buhnerkempe et al., 2015; Lloyd‐Smith et al., 2015; Plowright et al., 2017).

We parameterized our two‐ and three‐species models for Neotropical bat hosts, assuming that birth and natural death rates were similar and that interspecific competition was negligible among all co‐roosting species. However, future work could build upon the framework presented here by incorporating species‐specific demographics and explicit competition coefficients to provide further realism into models of cross‐species transmission. Demographic host traits pertaining to interspecific competitive pressures would ultimately shift population dynamics within a co‐roosting system over time, and thus alter intra‐ and interspecific transmission patterns and roost prevalence with phylogenetic relatedness at equilibrium. For example, varying hosts' natural birth and death rates could increase infection prevalence over time through a long‐lived host species (Anderson, 1982) or through larger birth pulses introducing more susceptible individuals into a population (Lloyd‐Smith et al., 2005; Peel et al., 2014). These models can serve as a starting point for future theory that may further expand upon the evolutionary barriers to transmission through social dynamics, creating competition between more or less social host species in a roost. Integrating within‐ and between‐host social dynamics (Altizer et al., 2003, Sah et al. 2018) into our phylogenetic framework could improve understanding species‐specific drivers of roost prevalence in co‐roosting environments. Further, simulating these models across empirical multi‐species contact networks would provide targeted model validation within co‐roosting systems of interest. Incorporating species‐specific traits that may impact both population size and interspecific interactions within a roost will be important next directions for estimating more specified cross‐species transmission dynamics within co‐roosting systems.

In addition to incorporating interspecific competition and social dynamics into future adaptations of the models presented here, these co‐roosting models could also be modified to explicitly incorporate vector‐borne or environmental transmission. For example, blood‐borne bacterial infections such as bartonellae and hemoplasmas have varying degrees of vector–host and host–host transmission (McKee et al., 2021; Millán et al., 2021). Future models could incorporate vector species with varying rates of host specificity while maintaining similar relationships between direct interspecific transmission and phylogenetic relatedness among hosts. Similarly, an additional compartment representing the concentration of a pathogen in the co‐roosting environment could also be integrated into this modeling framework, in a manner akin to that used for environmental transmission of avian influenza viruses (Wang et al., 2012). Such additions could integrate further epidemiological complexity and tease apart species‐specific contributions to multiple transmission routes and their outcomes through prevalence in the co‐roosting community.

Finally, the modeling framework we present here is representative of pathogen systems with relatively broad host ranges (i.e., herpesviruses and coronaviruses; Brito et al., 2021, Peck et al., 2015, Zhou et al., 2021), such that they are able to infect many host species due to highly conserved physiological host traits. However, for pathogen systems with narrower host ranges, it could be beneficial to adapt phylogenetic relatedness parameters to more specialized host traits. For example, this could be done by simply modifying the strength of exponential decay on interspecific transmission (λ), manipulating factorial values on a finer scale than presented here.

We contribute to growing efforts to better understand cross‐species transmission, particularly in relation to evolutionary barriers. A large body of previous theory has considered pathogen dynamics in multi‐host systems (Buhnerkempe et al., 2015; Cortez, 2021; Faust et al., 2017, 2018; Holt & Dobson, 2006; Roberts & Heesterbeek, 2018; Strauss et al., 2015), but the evolutionary underpinnings of cross‐species transmission remain poorly integrated into models. Recent work has explicitly modeled interspecific transmission as a proportional reduction of intraspecific transmission (Faust et al., 2018), and we expanded this approach to consider the well‐studied functional relationship (i.e., exponential decay) between phylogenetic distance and probability or frequency of cross‐species transmission (Albery et al., 2021; Faria et al., 2013; Huang et al., 2014; Longdon et al., 2011; Parker et al., 2015; Pedersen & Davies, 2009; Streicker et al., 2012; Willoughby et al., 2017). Here, we show that increasing phylogenetic similarity of co‐roosting hosts can facilitate the invasion of even poorly transmissible pathogens, supporting contexts in which multi‐host roosts could enhance infection risks. In all two‐ and three‐species SIRS and SILI models, we also found that roost prevalence maximized when species were most phylogenetically related, as supported by many empirical findings (Albery et al., 2021, Faria et al., 2013, Huang et al., 2014, Longdon et al., 2011, Parker et al., 2015, Pedersen & Davies, 2009, Streicker et al., 2012, Willoughby et al., 2017). However, we also found contexts where roost prevalence increased across distantly and unevenly related hosts, supporting increasing spillover risks due to frequent interactions between hosts from extreme spatial overlapping behaviors such as co‐roosting and gregariousness (Luis et al., 2015; Reluga et al., 2007). When parameterized for any one system, such models can generate realistic scenarios for how co‐roosting species contribute to community‐level infection prevalence and spillover risks. Future research modeling cross‐species transmission should also expand these frameworks by incorporating other barriers to pathogen transmission, such as social and foraging behaviors, multi‐host demographics, and multi‐host competition, in combination with the evolutionary barriers presented here.

## AUTHOR CONTRIBUTIONS

Molly C. Simonis and Daniel J. Becker equally contributed to the intellectual development of this manuscript; conceptualizing the idea; and developing models, code, and visualizations. Molly C. Simonis carried out numeric analyses and wrote the initial manuscript. Daniel J. Becker derived model analytics and provided feedback on all drafts of the manuscript.

## FUNDING INFORMATION

Molly C. Simonis was supported by an appointment to the Intelligence Community Postdoctoral Research Fellowship Program at University of Oklahoma administered by Oak Ridge Institute for Science and Education through an interagency agreement between the U.S. Department of Energy and the Office of the Director of National Intelligence. Daniel J. Becker was supported by the National Science Foundation (DBI 2515340, DEB 2508535) and the Edward Mallinckrodt, Jr. Foundation.

## CONFLICT OF INTEREST STATEMENT

The authors declare no conflicts of interest.

## Supporting information


Appendix S1.

Appendix S2.

## Data Availability

Data and code (Simonis, 2025) are available in Zenodo at https://doi.org/10.5281/zenodo.17726187.
