# Supplementary material for: A general framework for modeling pathogen transmission in co‐roosting host communities
Source: Ecology. 2026 Feb 20;107(2):e70326. doi: 10.1002/ecy.70326 (PMC12921667; doi:10.1002/ecy.70326)
Supplement: Supplementary file 1 — Appendix S1. [file ECY-107-e70326-s001.pdf]

Appendix S1

Supporting Information for

A general framework for modeling pathogen transmission in co-roosting host communities

Molly C. Simonis and Daniel J. Becker

Ecology

Model analysis

Two-host models

In the absence of infection, we can derive equilibria for our two-host co-roosting system by setting differential equations to zero. These correspond to the trivial equilibrium, in which both host species are extinct, and the disease-free equilibrium, in which both host species persist.

Extinction equilibrium: $(N_A^* = 0, N_B^* = 0)$
Disease-free equilibrium: $(N_A^* = K/2, N_B^* = K/2)$

Note that this particular disease-free equilibrium is appropriate for the parameterization specified in Table 2, in which both host species have equivalent starting population sizes (given that per-capita fecundity and natural mortality rates are equal among the two species).

We can evaluate the stability of each equilibrium by calculating the Jacobian matrix (J_{ij}), where the indexes describe the partial derivatives for the rate of change of variable *i* with respect to *j*. We use the Routh–Hurwitz criteria for stability, such that evaluating the Jacobian at any equilibrium must result in a negative trace (tr(*J*) < 0) and a positive determinant (*D* > 0).

$J_{1,1} = b_0 - 2b_1N_A - b_1N_B - \mu$
$J_{1,2} = -b_1N_A$
$J_{2,1} = -b_1N_B$
$J_{2,2} = b_0 - b_1N_A - 2b_1N_B - \mu$

For the extinction equilibrium, the Jacobian matrix simplifies as follows:

$J_{1,1} = b_0 - \mu$
$J_{1,2} = 0$
$J_{2,1} = 0$
$J_{2,2} = b_0 - \mu$

As such, the extinction equilibrium is stable if $b_0 < \mu$ and unstable if $b_0 > \mu$.

For the disease-free equilibrium, the Jacobian matrix simplifies as follows:

$$J_{1,1} = -\frac{b_1K}{2}$$

$$J_{1,2} = -\frac{b_1 K}{2}$$
$$J_{2,1} = -\frac{b_1 K}{2}$$
$$J_{2,2} = -\frac{b_1 K}{2}$$

As such, tr(*J*) < 0 when $b_0 > \mu$, while tr(*J*) > 0 when $b_0 < \mu$.
The determinant here factorizes as zero, such that the demographic symmetry between hosts results in a neutrally stable, rather than asymptomatically stable, disease-free equilibrium.

In the presence of acute infection with waning immunity (i.e., the SIRS model), the conditions for stability of the extinction and disease-free equilibria are the same, provided the pathogen does not invade. Pathogen invasion occurs when the basic reproductive number (R_0) exceeds 1, resulting in an endemic equilibrium (i.e., at least one infected class is non-zero). This equilibrium is difficult to solve analytically, such that we rely on solving the model numerically and instead derive R_0 using the next-generation matrix. Here, we first, we identify the terms in the two-host SIRS system of equations resulting in gains and losses to infected classes (I_A and I_B):

I_A gains = $\beta S_A I_A + \theta S_A I_B$
I_B gains = $\beta S_B I_B + \theta S_B I_A$
I_A losses = $(\mu + \gamma) I_A$
I_B losses = $(\mu + \gamma) I_B$

Then we calculate the matrix *F*, whose entries are the partial derivatives of the gain terms with respect to each infected host type:

$$F_{1,1} = \beta S_A$$
$$F_{1,2} = \theta S_A$$
$$F_{2,1} = \theta S_B$$
$$F_{2,2} = \beta S_B$$

We repeat this process for the matrix *V*, for the equivalent partial derivatives of loss terms:

$$V_{1,1} = \mu + \gamma$$
$$V_{1,2} = 0$$
$$V_{2,1} = 0$$
$$V_{2,2} = \mu + \gamma$$

We then calculate the matrix *G* as FV^{-1}:

$$G_{1,1} = \frac{\beta S_A}{\mu + \gamma}$$
$$G_{1,2} = \frac{\theta S_A}{\mu + \gamma}$$
$$G_{2,1} = \frac{\theta S_B}{\mu + \gamma}$$
$$G_{2,2} = \frac{\beta S_B}{\mu + \gamma}$$

The dominant eigenvalue here is R_0, which we evaluate at the disease-free equilibrium:

$$R_0 = \frac{K(\beta + \theta)}{2(\mu + \gamma)}$$

We can then express R_0 using the expression for θ in the two-host co-roosting SIRS system:

$$R_0 = \frac{K\beta(1 + e^{(1-\psi)\lambda})}{2(\mu + \gamma)}$$

We can repeat this process for a chronic infection with reactivation (i.e., the SILI model), for which both the I classes (I_A and I_B) and L classes (L_A and L_B) contribute to infection, but *new* infections are only gained by the I classes.

I_A gains = $\beta S_A I_A + \theta S_A I_B$
L_A gains = 0
I_B gains = $\beta S_B I_B + \theta S_B I_A$
L_B gains = 0

I_A losses = $(\mu + \gamma)I_A - \omega L_A$
L_A losses = $(\mu + \omega)L_A - \gamma I_A$
I_B losses = $(\mu + \gamma)I_B - \omega L_B$
L_B losses = $(\mu + \omega)L_B - \gamma I_B$

This results in a four-by-four F matrix (I_A, L_A, I_B, L_B):

$F_{1,1} = \beta S_A$
$F_{1,2} = 0$
$F_{1,3} = \theta S_A$
$F_{1,4} = 0$
$F_{2,1} = 0$
$F_{2,2} = 0$
$F_{2,3} = 0$
$F_{2,4} = 0$
$F_{3,1} = \theta S_B$
$F_{3,2} = 0$
$F_{3,3} = \beta S_B$
$F_{3,4} = 0$
$F_{4,1} = 0$
$F_{4,2} = 0$
$F_{4,3} = 0$
$F_{4,4} = 0$

We similarly derive an equivalent four-by-four V matrix:

$V_{1,1} = \mu + \gamma$
$V_{1,2} = -\omega$

$V_{1,3} = 0$
$V_{1,4} = 0$
$V_{2,1} = -\gamma$
$V_{2,2} = \mu + \omega$
$V_{2,3} = 0$
$V_{2,4} = 0$
$V_{3,1} = 0$
$V_{3,2} = 0$
$V_{3,3} = \mu + \gamma$
$V_{3,4} = -\omega$
$V_{4,1} = 0$
$V_{4,2} = 0$
$V_{4,3} = -\gamma$
$V_{4,4} = \mu + \omega$

We again calculate the matrix *G* as FV^{-1}:

$$G_{1,1} = \frac{\beta S_A(\mu + \omega)}{\mu(\mu + \gamma + \omega)}$$
$$G_{1,2} = \frac{\beta S_A \omega}{\mu(\mu + \gamma + \omega)}$$
$$G_{1,3} = \frac{\theta S_A(\mu + \omega)}{\mu(\mu + \gamma + \omega)}$$
$$G_{1,4} = \frac{\theta S_A \omega}{\mu(\mu + \gamma + \omega)}$$
$G_{2,1} = 0$
$G_{2,2} = 0$
$G_{2,3} = 0$
$G_{2,4} = 0$
$$G_{3,1} = \frac{\theta S_B(\mu + \omega)}{\mu(\mu + \gamma + \omega)}$$
$$G_{3,2} = \frac{\theta S_B \omega}{\mu(\mu + \gamma + \omega)}$$
$$G_{3,3} = \frac{\beta S_B(\mu + \omega)}{\mu(\mu + \gamma + \omega)}$$
$$G_{3,4} = \frac{\beta S_B \omega}{\mu(\mu + \gamma + \omega)}$$
$G_{4,1} = 0$
$G_{4,2} = 0$
$G_{4,3} = 0$
$G_{4,4} = 0$

Given the block-sparse nature of the *G* matrix, we can simplify this to an effective two-by-two *G* matrix that instead only contains the contributions of the infectious host compartments:

$$G_{1,1} = \frac{\beta S_A(\mu + \omega)}{\mu(\mu + \gamma + \omega)}$$

$$G_{1,2} = \frac{\theta S_A(\mu + \omega)}{\mu(\mu + \gamma + \omega)}$$
$$G_{2,1} = \frac{\theta S_B(\mu + \omega)}{\mu(\mu + \gamma + \omega)}$$
$$G_{2,2} = \frac{\beta S_B(\mu + \omega)}{\mu(\mu + \gamma + \omega)}$$

Deriving the dominant eigenvalue of this effective *G* matrix at the disease-free equilibrium, with the full expression for θ, gives us the R_0 for the two-host co-roosting SILI system:

$$R_0 = \frac{K\beta(1 + e^{(1-\psi)\lambda})(\mu + \omega)}{2\mu(\mu + \gamma + \omega)}$$

Three-host models

As in our two-host models, we can derive the equilibria under three co-roosting species in the absence of infection. These again correspond to the trivial extinction equilibrium and the disease-free equilibrium (under which demographic rates and starting conditions are the same).

Extinction equilibrium: $(N_A^* = 0, N_B^* = 0, N_C^* = 0)$
Disease-free equilibrium: $(N_A^* = K/3, N_B^* = K/3, N_C^* = K/3)$

Evaluating the stability of these equilibria results in the following three-by-three Jacobian matrix:

$$J_{1,1} = b_0 - 2b_1N_A - b_1N_B - b_1N_C - \mu$$
$$J_{1,2} = -b_1N_A$$
$$J_{1,3} = -b_1N_A$$
$$J_{2,1} = -b_1N_B$$
$$J_{2,2} = b_0 - b_1N_A - 2b_1N_B - b_1N_C - \mu$$
$$J_{2,3} = -b_1N_B$$
$$J_{3,1} = -b_1N_C$$
$$J_{3,2} = -b_1N_C$$
$$J_{3,3} = b_0 - b_1N_A - b_1N_B - 2b_1N_C - \mu$$

For the extinction equilibrium, the Jacobian matrix simplifies as follows:

$$J_{1,1} = b_0 - \mu$$
$$J_{1,2} = 0$$
$$J_{1,3} = 0$$
$$J_{2,1} = 0$$
$$J_{2,2} = b_0 - \mu$$
$$J_{2,3} = 0$$
$$J_{3,1} = 0$$
$$J_{3,2} = 0$$
$$J_{3,3} = b_0 - \mu$$

As such, the extinction equilibrium is again stable if $b_0 < \mu$ and unstable if $b_0 > \mu$.

For the disease-free equilibrium, similar to that in the two-host model, *J* simplifies as follows:

$$J_{1,1} = -\frac{b_1 K}{3}$$
$$J_{1,2} = -\frac{b_1 K}{3}$$
$$J_{1,3} = -\frac{b_1 K}{3}$$
$$J_{2,1} = -\frac{b_1 K}{3}$$
$$J_{2,2} = -\frac{b_1 K}{3}$$
$$J_{2,3} = -\frac{b_1 K}{3}$$
$$J_{3,1} = -\frac{b_1 K}{3}$$
$$J_{3,2} = -\frac{b_1 K}{3}$$
$$J_{3,3} = -\frac{b_1 K}{3}$$

As such, tr(*J*) < 0 when $b_0 > \mu$, while tr(*J*) > 0 when $b_0 < \mu$.
The determinant again factorizes as zero, such that the demographic symmetry between hosts again results in a neutrally stable, rather than asymptomatically stable, disease-free equilibrium.

For both the SIRS and SILI models with three co-roosting species, we again use the next-generation matrix method to derive the respective R_0 expressions.

For the SIRS model, we again identify gains and losses to the infected classes (I_A, I_B, and I_C):

I_A gains = $\beta S_A I_A + \theta_{AB} S_A I_B + \theta_{AC} S_A I_C$
I_B gains = $\beta S_B I_B + \theta_{BA} S_B I_A + \theta_{BC} S_B I_C$
I_C gains = $\beta S_C I_C + \theta_{CA} S_C I_A + \theta_{CB} S_C I_B$
I_A losses = $(\mu + \gamma) I_A$
I_B losses = $(\mu + \gamma) I_B$
I_C losses = $(\mu + \gamma) I_C$

As in the analysis of the two-host SIRS model, we compute the three-by-three *K* and *V* matrices to obtain $G = FV^{-1}$, facilitated by the uniform diagonal of *V*:

$$G_{1,1} = \frac{\beta S_A}{\mu + \gamma}$$
$$G_{1,2} = \frac{\theta_{AB} S_A}{\mu + \gamma}$$
$$G_{1,3} = \frac{\theta_{AC} S_A}{\mu + \gamma}$$
$$G_{2,1} = \frac{\theta_{BA} S_B}{\mu + \gamma}$$

$$G_{2,2} = \frac{\beta S_B}{\mu + \gamma}$$

$$G_{2,3} = \frac{\theta_{BC} S_B}{\mu + \gamma}$$

$$G_{3,1} = \frac{\theta_{CA} S_C}{\mu + \gamma}$$

$$G_{3,2} = \frac{\theta_{CB} S_C}{\mu + \gamma}$$

$$G_{3,3} = \frac{\beta S_C}{\mu + \gamma}$$

Deriving the dominant eigenvalue of the *G* matrix at the disease-free equilibrium gives us the R_0 for the three-host co-roosting SIRS system. Given the complexity of the resulting expression, it is analytically more tractable to here work with the θ terms for inter-specific transmission.

We first define *P*, the sum of the reciprocal transmission among host species pairs:

$$P = \frac{(\theta_{AB}\theta_{BA}) + (\theta_{AC}\theta_{CA}) + (\theta_{BC}\theta_{CB})}{\beta^2}$$

We next define *C*, the sum of directional transmission cycles between the three host species:

$$C = \frac{(\theta_{AB}\theta_{BC}\theta_{CA}) + (\theta_{AC}\theta_{CB}\theta_{BA})}{\beta^3}$$

Note that because θ for any two species pairs is equal (i.e., pairwise phylogenetic similarity), *P* and *C* reduce to the following expressions:

$$P = \frac{\theta_{AB}^2 + \theta_{BC}^2 + \theta_{AC}^2}{\beta^2}$$

$$C = \frac{2(\theta_{AB}\theta_{AC}\theta_{BC})}{\beta^3}$$

Stemming from the discriminant of the cubic equation, we then derive Δ:

$$\Delta = \left(\frac{C}{2}\right)^2 - \left(\frac{P}{3}\right)^3$$

Lastly, we then derive Λ as the largest root of the cubic equation:

$$\Lambda = 1 + \sqrt[3]{\frac{C}{2} + \sqrt{\Delta}} + \sqrt[3]{\frac{C}{2} - \sqrt{\Delta}}$$

With these terms, we can then define R_0:

$$R_0 = \frac{K\beta}{3(\mu + \gamma)}\Lambda$$

For the three-host SILI model, we again identify gains and losses to the infected (I_A, I_B, and I_C) and latent (L_A, L_B, and L_C) classes:

I_A gains = $\beta S_A I_A + \theta_{AB} S_A I_B + \theta_{AC} S_A I_C$
L_A gains = 0
I_B gains = $\beta S_B I_B + \theta_{BA} S_B I_A + \theta_{BC} S_B I_C$
L_B gains = 0
I_C gains = $\beta S_C I_C + \theta_{CA} S_C I_A + \theta_{CB} S_C I_B$
L_C gains = 0
I_A losses = $(\mu + \gamma) I_A - \omega L_A$
L_A losses = $(\mu + \omega) L_A - \gamma I_A$
I_B losses = $(\mu + \gamma) I_B - \omega L_B$
L_B losses = $(\mu + \omega) L_B - \gamma I_B$
I_C losses = $(\mu + \gamma) I_C - \omega L_C$
L_C losses = $(\mu + \omega) L_C - \gamma I_C$

This results in a six-by-six *F* matrix (I_A, L_A, I_B, L_B, I_C, L_C):

$F_{1,1} = \beta S_A$
$F_{1,2} = 0$
$F_{1,3} = \theta_{AB} S_A$
$F_{1,4} = 0$
$F_{1,5} = \theta_{AC} S_A$
$F_{1,6} = 0$
$F_{2,1} = 0$
$F_{2,2} = 0$
$F_{2,3} = 0$
$F_{2,4} = 0$
$F_{2,5} = 0$
$F_{2,6} = 0$
$F_{3,1} = \theta_{BA} S_B$
$F_{3,2} = 0$
$F_{3,3} = \beta S_B$
$F_{3,4} = 0$
$F_{3,5} = \theta_{BC} S_B$
$F_{3,6} = 0$
$F_{4,1} = 0$
$F_{4,2} = 0$
$F_{4,3} = 0$
$F_{4,4} = 0$
$F_{4,5} = 0$
$F_{4,6} = 0$
$F_{5,1} = \theta_{CA} S_C$
$F_{5,2} = 0$
$F_{5,3} = \theta_{CB} S_C$
$F_{5,4} = 0$
$F_{5,5} = \beta S_C$
$F_{5,6} = 0$

$F_{6,1} = 0$
$F_{6,2} = 0$
$F_{6,3} = 0$
$F_{6,4} = 0$
$F_{6,5} = 0$
$F_{6,6} = 0$

We similarly derive an equivalent six-by-six *V* matrix:

$V_{1,1} = \mu + \gamma$
$V_{1,2} = -\omega$
$V_{1,3} = 0$
$V_{1,4} = 0$
$V_{1,5} = 0$
$V_{1,6} = 0$
$V_{2,1} = -\gamma$
$V_{2,2} = \mu + \omega$
$V_{2,3} = 0$
$V_{2,4} = 0$
$V_{2,5} = 0$
$V_{2,6} = 0$
$V_{3,1} = 0$
$V_{3,2} = 0$
$V_{3,3} = \mu + \gamma$
$V_{3,4} = -\omega$
$V_{3,5} = 0$
$V_{3,6} = 0$
$V_{4,1} = 0$
$V_{4,2} = 0$
$V_{4,3} = -\gamma$
$V_{4,4} = \mu + \omega$
$V_{4,5} = 0$
$V_{4,6} = 0$
$V_{5,1} = 0$
$V_{5,2} = 0$
$V_{5,3} = 0$
$V_{5,4} = 0$
$V_{5,5} = \mu + \gamma$
$V_{5,6} = -\omega$
$V_{6,1} = 0$
$V_{6,2} = 0$
$V_{6,3} = 0$
$V_{6,4} = 0$
$V_{6,5} = -\gamma$
$V_{6,6} = \mu + \omega$

As with our two-host SILI model, we leverage the block-sparse nature of these matrices to generate an effective three-by-three *G* matrix, evaluated at the disease-free equilibrium:

$$G_{1,1} = \frac{K\beta(\mu+\omega)}{3\mu(\mu+\gamma+\omega)}$$
$$G_{1,2} = \frac{K\theta_{AB}(\mu+\omega)}{3\mu(\mu+\gamma+\omega)}$$
$$G_{1,3} = \frac{K\theta_{AC}(\mu+\omega)}{3\mu(\mu+\gamma+\omega)}$$
$$G_{2,1} = \frac{K\theta_{BA}(\mu+\omega)}{3\mu(\mu+\gamma+\omega)}$$
$$G_{2,2} = \frac{K\beta(\mu+\omega)}{3\mu(\mu+\gamma+\omega)}$$
$$G_{2,3} = \frac{K\theta_{BC}(\mu+\omega)}{3\mu(\mu+\gamma+\omega)}$$
$$G_{3,1} = \frac{K\theta_{CA}(\mu+\omega)}{3\mu(\mu+\gamma+\omega)}$$
$$G_{3,2} = \frac{K\theta_{CB}(\mu+\omega)}{3\mu(\mu+\gamma+\omega)}$$
$$G_{3,3} = \frac{K\beta(\mu+\omega)}{3\mu(\mu+\gamma+\omega)}$$

We can leverage the same expressions for *P*, *C*, Δ, and Λ to then derive the three-host, SILI R_0:

$$R_0 = \frac{K\beta(\mu+\omega)}{3\mu(\mu+\gamma+\omega)}\Lambda$$
