# Supplementary material for: A general framework for modeling pathogen transmission in co‐roosting host communities
Source: Ecology. 2026 Feb 20;107(2):e70326. doi: 10.1002/ecy.70326 (PMC12921667; doi:10.1002/ecy.70326)
Supplement: Supplementary file 2 — Appendix S2. [file ECY-107-e70326-s002.pdf]

<u>Appendix S2</u>

Supporting Information for

A general framework for modeling pathogen transmission in co-roosting host communities

Molly C. Simonis and Daniel J. Becker

Ecology

Supplementary Figures

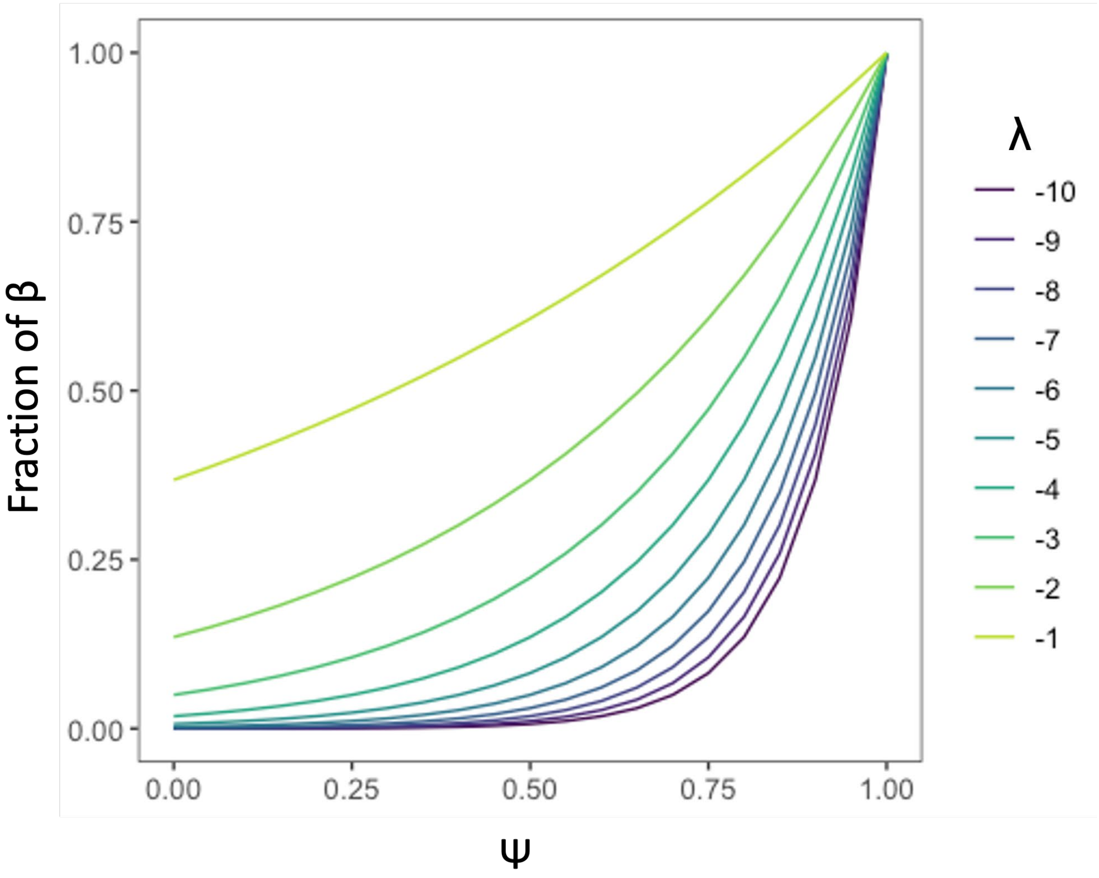

Figure S1. Examples of functional relationships between host phylogenetic similarity and pathogen transmission between host species. The fraction of intraspecific transmission (β) is determined by the function $e^{(1-\Psi)\lambda}$, where Ψ is the correlation coefficient of phylogenetic relatedness between species and λ is the shape of the curve. Colors represent different λ values.

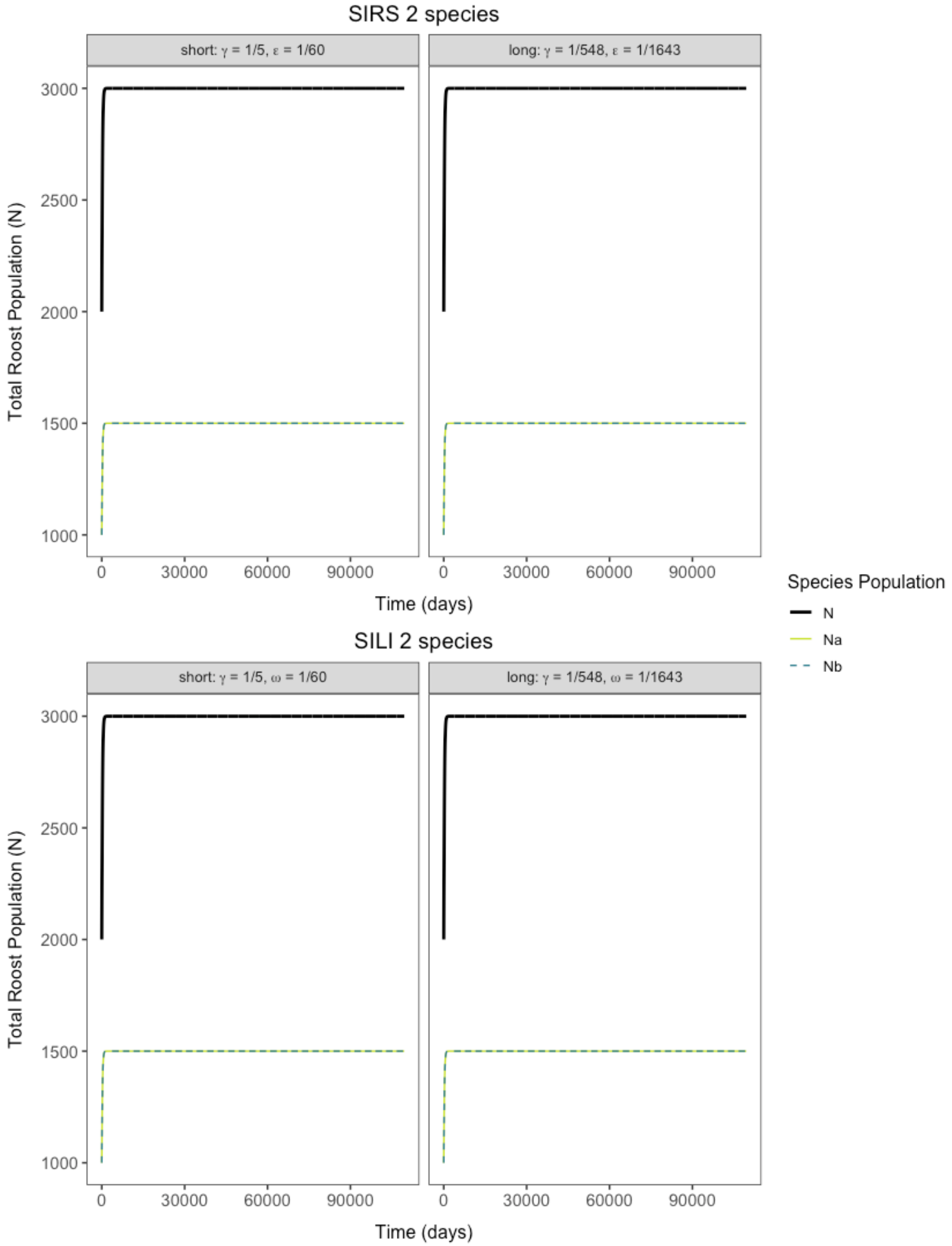

Figure S2. Disease-free time series examples of two-species SIRS and SILI models under the lowest interspecific transmission (β = 0.0005), moderate effects of phylogenetic distance on interspecific transmission (λ = -5), and varying infectious periods (1/γ) and immunity (1/ε; SIRS only) or latent periods (1/ω; SILI only). Species in each example are 50% related to one another (Ψ). Models were run over 300 years. Colors and line-types represent each species population

or the total roost population (thick black line) over time. Note that individual species populations are the same over time for each species.

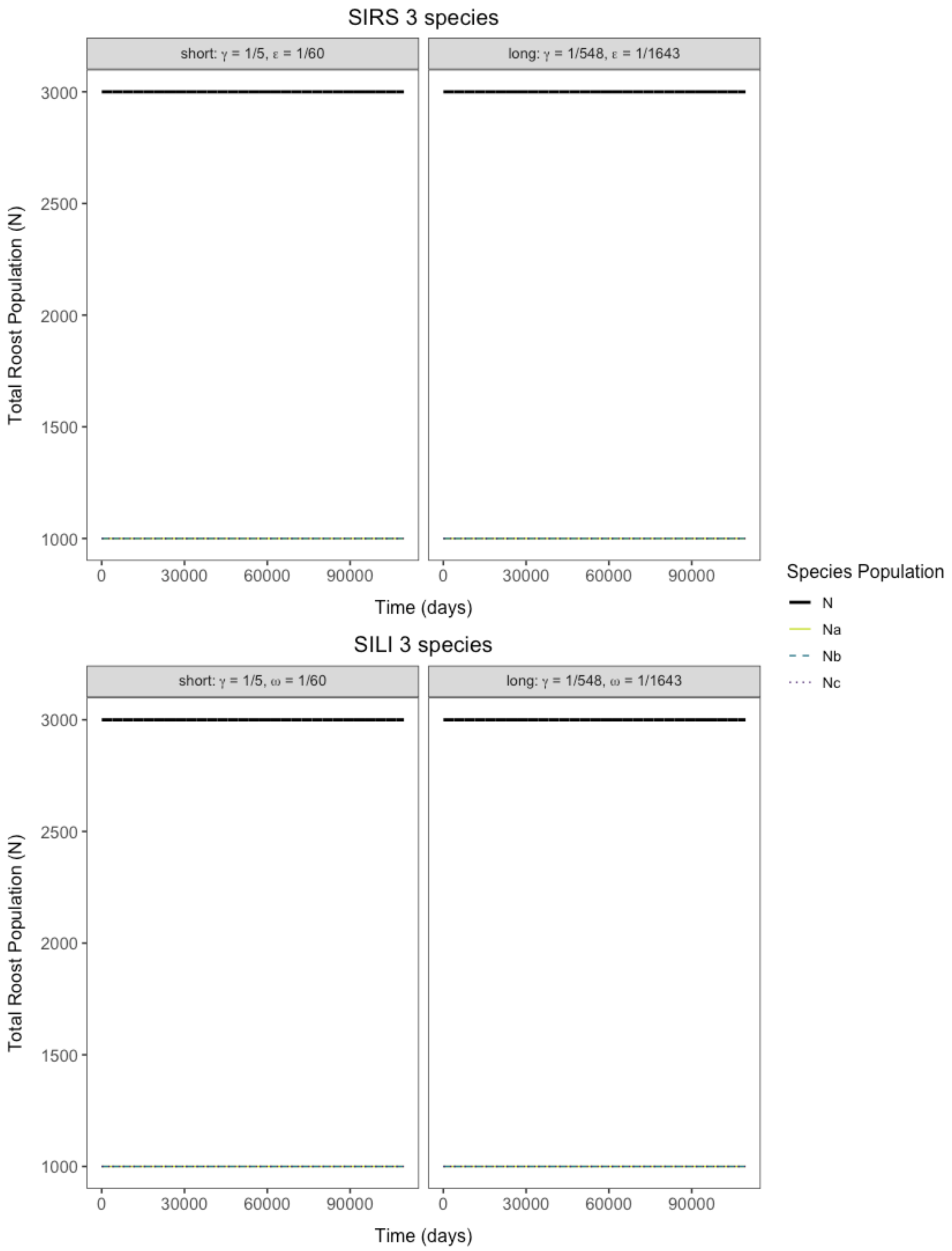

Figure S3. Disease-free time series examples of three-species SIRS and SILI models under the lowest interspecific transmission (β = 0.0005), moderate effects of phylogenetic distance on interspecific transmission (λ = -5), and varying infectious periods (1/γ) and immunity (1/ε; SIRS only) or latent periods (1/ω; SILI only). All species in each example are 50% related to one another (Ψ). Lines represent the total population (N) or species-specific population within the roost (N_A or N_B). Models were run over 300 years. Colors and line-types represent each species

population or the total roost population (thick black line) over time. Note that individual species populations are the same over time for each species.

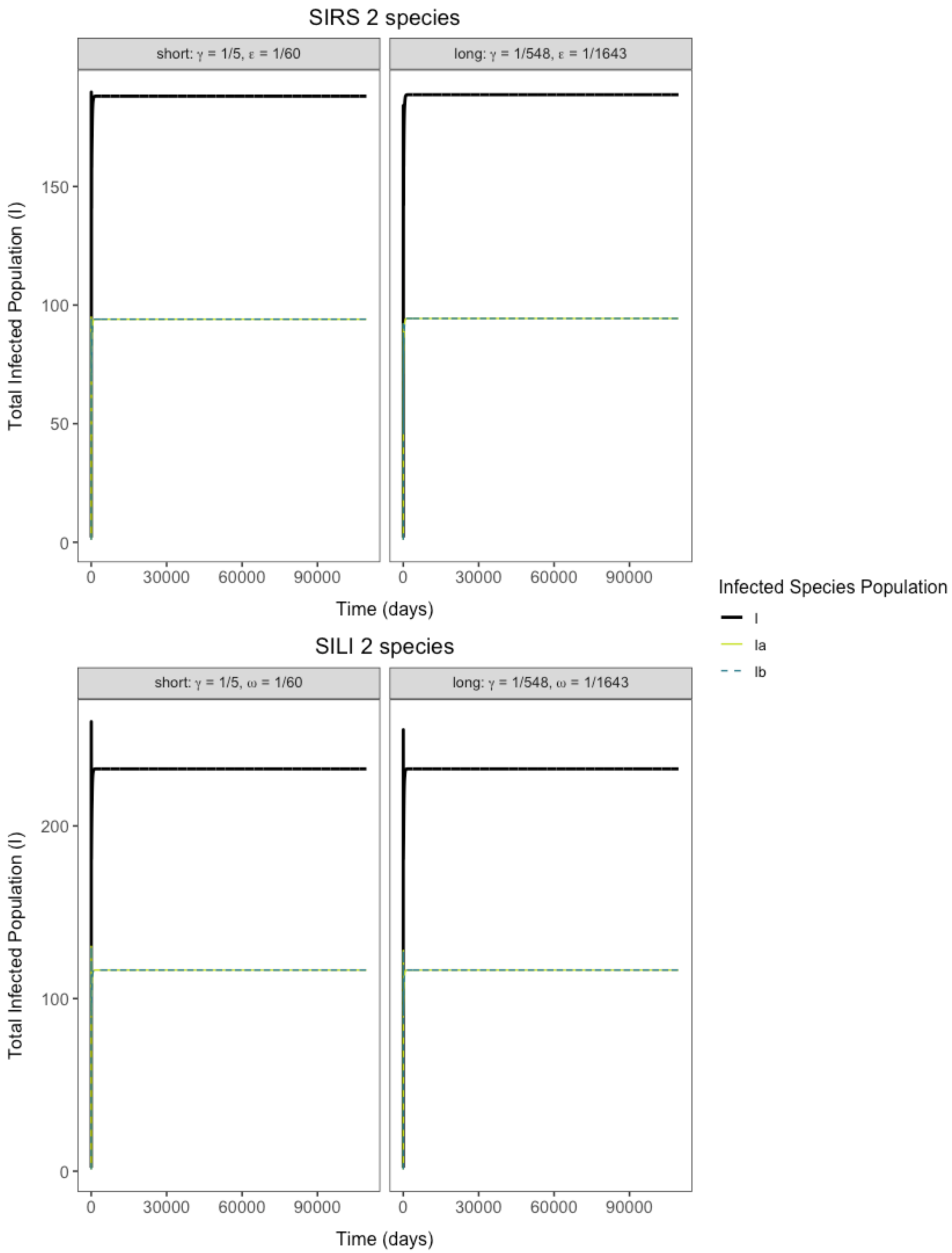

Figure S4. Time series examples of two-species SIRS and SILI models under the lowest interspecific transmission (β = 0.0005), moderate effects of phylogenetic distance on interspecific transmission (λ = -5), and varying infectious periods (1/γ) and immunity (1/ε; SIRS only) or latent periods (1/ω; SILI only). Species in each example are 50% related to one another (Ψ). Models were run over 300 years, and started with one infected individual and 999 susceptible individuals in each species population. Colors and line-types represent each

species’ infectious populations or the total infectious population within the roost (thick black line) over time. Note that individual infectious species populations are the same over time for each species.

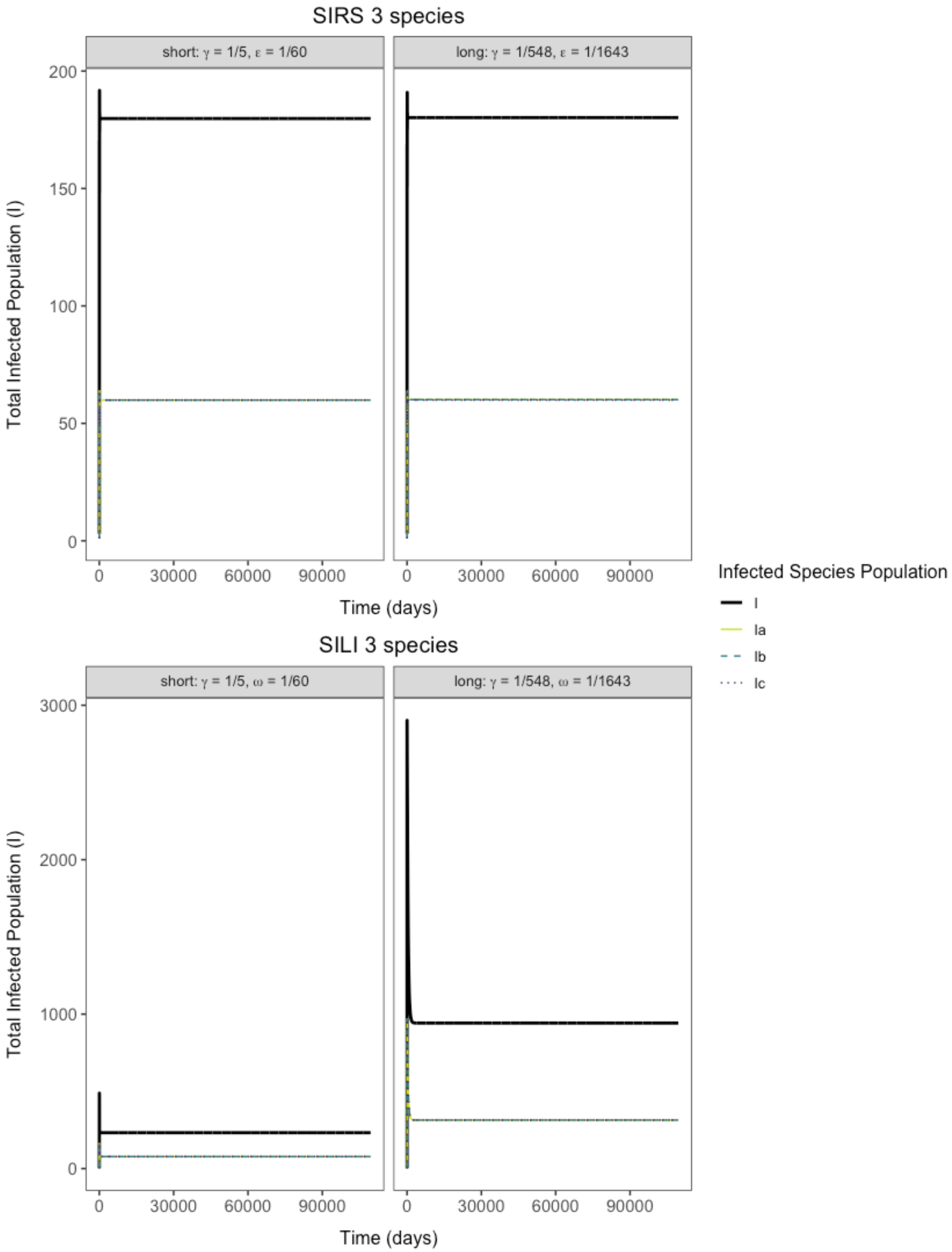

Figure S5. Time series examples of three-species SIRS and SILI models under the lowest interspecific transmission (β = 0.0005), moderate effects of phylogenetic distance on interspecific transmission (λ = -5), and varying infectious periods (1/γ) and immunity (1/ε; SIRS only) or latent periods (1/ω; SILI only). Species in each example are 50% related to one another (Ψ). Models were run over 300 years, and started with one infected individual and 999 susceptible individuals in each species population. Colors and line-types represent each

species’ infectious populations or the total infectious population within the roost (thick black line) over time. Note that individual infectious species populations are the same over time for each species.
